# Arginine methylation of caspase-8 controls life/death decisions in extrinsic apoptotic networks

**DOI:** 10.1038/s41388-024-03049-6

**Published:** 2024-05-10

**Authors:** Fabian Wohlfromm, Nikita V. Ivanisenko, Sabine Pietkiewicz, Corinna König, Kamil Seyrek, Thilo Kähne, Inna N. Lavrik

**Affiliations:** 1 Translational Inflammation Research, Medical Faculty, Center of Dynamic Systems (CDS), Otto von Guericke University, 39106 Magdeburg, Germany; 2 Institute of Experimental Internal Medicine, Medical Faculty, Otto von Guericke University, 39120 Magdeburg, Germany

**Keywords:** Apoptosis, Proteomics, Methylation

## Abstract

Procaspase-8 is a key mediator of death receptor (DR)-mediated pathways. Recently, the role of post-translational modifications (PTMs) of procaspase-8 in controlling cell death has received increasing attention. Here, using mass spectrometry screening, pharmacological inhibition and biochemical assays, we show that procaspase-8 can be targeted by the PRMT5/RIOK1/WD45 methylosome complex. Furthermore, two potential methylation sites of PRMT5 on procaspase-8, R233 and R435, were identified in silico. R233 and R435 are highly conserved in mammals and their point mutations are among the most common mutations of caspase-8 in cancer. The introduction of mutations at these positions resulted in inhibitory effects on CD95L-induced caspase-8 activity, effector caspase activation and apoptosis. In addition, we show that procaspase-8 can undergo symmetric di-methylation. Finally, the pharmacological inhibition of PRMT5 resulted in the inhibitory effects on caspase activity and apoptotic cell death. Taken together, we have unraveled the additional control checkpoint in procaspase-8 activation and the arginine methylation network in the extrinsic apoptosis pathway.

## Introduction

Apoptosis is a programme of cell death, which is essential for all multicellular organisms [1]. The death receptor (DR) family is a subfamily of the tumor necrosis factor receptor (TNFR)-superfamily. CD95/Fas is a member of the DR family [1, 2]. Activation of CD95 initiates the extrinsic apoptosis pathway *via* the formation of a death-inducing signaling complex (DISC), comprising CD95, FADD, procaspases-8, -10 and c-FLIPs [3]. The DISC serves as a major platform for procaspase-8 activation, which subsequently triggers an apoptotic cascade.

Procaspase-8 is an initiator caspase of DR-induced apoptosis and its activation defines the onset of cell death. Procaspase-8 contains two death effector domains (DEDs) at its N-terminus: DED1 and DED2 followed by two protease domains: p10 and p18 at the C-terminal region [4]. Autocatalytic activation of procaspase-8 occurs upon its dimerisation, leading to the formation of the active caspase-8 heterotetramer p10_2_-p18_2_ [5]. Two major procaspase-8 isoforms have been described: procaspase-8a and -8b, p55 and p53, respectively [6]. These two isoforms differ in a small region of 2 kDa after DED2, which is absent in procaspase-8b, p53. The main functional domains of these two isoforms are identical: DED1, DED2, p18 and p10 [7]. Interactions of procaspase-8 DEDs at the DISC lead to the assembly of DED filaments [8–11]. The filaments serve as a platform for dimerisation and subsequent activation of procaspase-8, which in turn triggers apoptosis.

Recent findings have revealed a role for post-translational modifications (PTMs) such as phosphorylation, ubiquitylation, SUMOylation, parylation and nitrosylation in regulating procaspase-8 interactions and activation [4, 12–18]. These modifications help to maintain the correct conformation of procaspase-8 in DED filaments, which is required for efficient caspase-8 activation and overcoming the threshold for apoptosis induction [19–21]. However, the detailed roles of PTMs in procaspase-8 activation are still largely unknown. Furthermore, some PTMs such as methylation and acetylation of procaspase-8 have not been described yet.

Methylation of DNA, RNA and proteins plays a pivotal role in cellular processes [22–25]. While the methylation of nucleic acids has been extensively studied to date, protein methylation is only beginning to be elucidated [26, 27]. This process is mediated by protein methyltransferases (PRMTs), which add methyl groups to their target proteins. PRMT5 is one of the eleven human protein methyltransferases described to date, and specifically methylates arginine residues. PRMT5 substrates include the spliceosome, histone and ribosome assembly complexes [22, 28–32]. The methylosome complex of PRMT5 includes MEP50/WD45/WDR77 (WD repeat-containing protein), which is essential for its methylation function [29]. The core complex formed by PRMT5/WD45 recruits one of three adaptor proteins. One of these is RIOK1, an atypical serine/threonine kinase that acts as a scaffold for the PRMT5 methylosome [29]. In addition to RIOK1, two other adaptor proteins: pICIn (chloride channel nucleotide sensitive 1 A) and COPR5 (coordinator of PRMT5 and differentiation stimulator) constitute distinct PRMT5 complexes [28, 30, 31]. The RIOK1-dependent complex is localised in the cytosol, whereas the pICIn- and COPR5-mediated complexes are detected in the nucleus [28, 29].

Deregulation of procaspase-8 expression and mutations in procaspase-8 have been reported in several tumor types [33, 34]. This highlights the importance of understanding the regulatory network of procaspase-8 in cancer cells and the role of PTMs in its activation. Furthermore, there are still a number of unresolved molecular mechanisms concerning the role of procaspase-8 PTMs in cell fate. This knowledge is particularly important for understanding the regulation of DED networks and for developing new treatment strategies in cancer.

In this study, we investigated the role of methylation in activation of caspase-8. Using pharmacological targeting and the introduction of mutations we have found that methylation of procaspase-8 may be required for its activation. This work may also shed light on the additional regulation of procaspase-8 activation in the context of diseases that are connected to deregulation of DR signaling.

## Results

### Mass spectrometry screening identifies the core components of the PRMT5 complex in the caspase-8 interactome

**Fig. 1:**
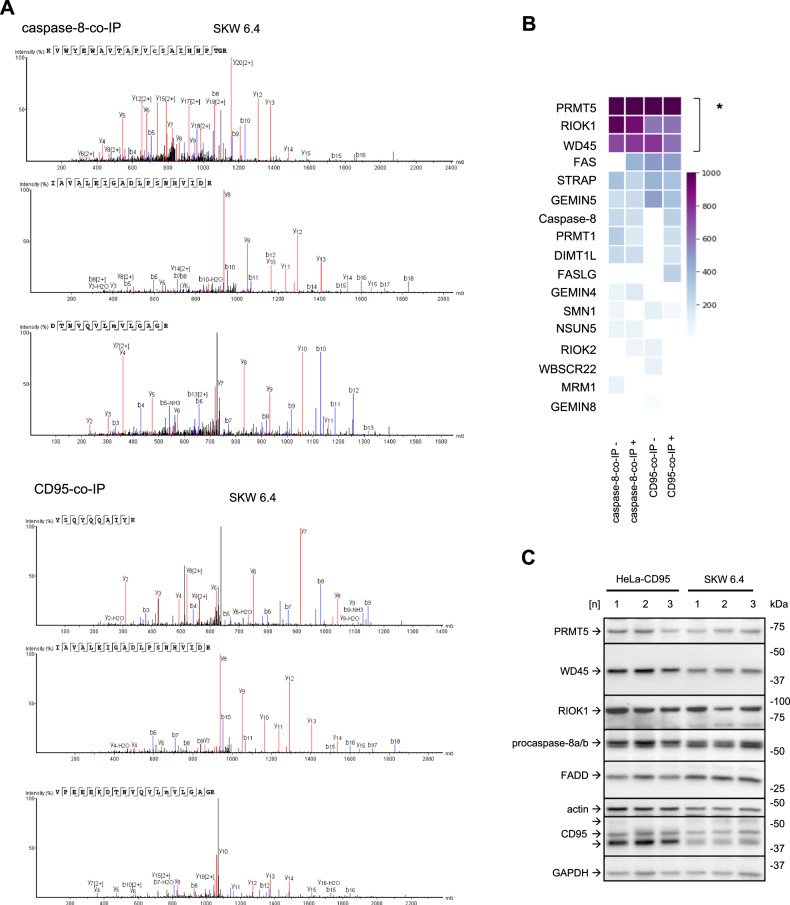
Mass spectrometry analysis identifies the core components of the PRMT5 complex in caspase-8 complexes by mass spectrometry. **A** Mass spectrometry identification of unique proteins in caspase-8 co-Immunoprecipitations (caspase-8-co-IP) and CD95-co-IPs. Mass spectra corresponding to the analysis of PRMT5 in caspase-8-co-IP (upper part) or CD95-co-IP (lower part) after CD95L treatment are shown. Three independent replicates are presented. The SKW 6.4 cells were stimulated for 15 minutes with 166 ng/ml of CD95L. In these experiments, anti-caspase-8 and anti-CD95 (anti-APO-1) antibodies were immobilized at the beads, followed by co-immunoprecipitation (co-IP). The co-IPs from untreated and CD95L-treated SKW 6.4 cells were subjected to mass spectrometry analysis. **B** Results of mass spectrometry analysis for caspase-8-co-IP and CD95-co-IP experiments. The median of the unique peptides from three independent experiments is shown for each protein for CD95L-stimulated (+) and non-stimulated (–) samples. The scoring indicates the increase in unique peptides identified for each protein. Proteins with statistically significantly higher scores compared to other identified proteins are highlighted (* - *p*-value < 0.05). The statistical significance was calculated with a one-sample bootstrap method. Only proteins of the human methylome and core DISC components are shown. **C** Expression of indicated proteins in HeLa-CD95 and SKW 6.4 cells was analysed by Western Blot using the corresponding antibodies. Shown are three different cell passage numbers (1, 2, 3) indicated as [n]. *co-IP* co-immunoprecipitation; beads-only pulldown, [n], cell passage number.

Mass spectrometry analysis of CD95-co-immunoprecipitation (CD95-co-IP) and caspase-8-co-IP from CD95L-stimulated B lymphoblastoid SKW 6.4 cells has identified the presence of the core components of the PRMT5 methylosome complex: PRMT5, WD45 and RIOK1 in these co-IPs (Fig. 1A, B; Supplementary Fig. 1A–C). These three proteins were detected with high confidence in both the CD95-co-IP and the caspase-8-co-IP (Fig. 1A, B; Supplementary Fig. 1A–C). Caspase-8 and CD95/Fas were found in CD95-co-IP and caspase-8-co-IPs, respectively, only upon CD95L stimulation, indicating the specificity of the DISC formation (Fig. 1B). The other core component of the DISC, FADD, was not detected in this assay. FADD is very difficult to detect in the mass spectrometry analysis of the DISC due to the lower abundance of FADD compared to caspase-8 in this complex, as shown previously [8, 9]. The other proteins from the human methylome besides PRMT5, WD45 and RIOK1 were also found in this mass spectrometry analysis, but with the lower scores (Fig. 1B).

PRMT5, WD45 and RIOK1 are expressed in different cancer cell lines such as cervical cancer HeLa-CD95 [35] and B lymphoblastoid SKW 6.4 cells (Fig. 1C). The PRMT5 methylosome complexes have been reported to be localized in the nucleus as well as in the cytosol [29]. However, the PRMT5 complex that comprises RIOK1 is known to reside in the cytosol. Hence, we next tested whether PRMT5, WD45 and RIOK1 could be found in the cytosolic fraction after cell fractionation. Fractionation of SKW 6.4 cells showed that PRMT5, WD45 and RIOK1 were detected in both nuclear and cytosolic fractions with and without CD95L stimulation, suggesting a possible involvement of these proteins in cytosolic cell death pathways (Supplementary Fig. 1D).

The results of the mass spectrometry screening were validated by co-IPs followed by Western Blot analysis (Supplementary Fig. 2A, B). In these experiments, SKW 6.4 cells were stimulated with CD95L, followed by co-IP with anti-caspase-8 antibodies. These experiments elucidated the presence of PRMT5, WD45 and RIOK1 in caspase-8-co-IPs in SKW 6.4 cells (Supplementary Fig. 2A, B). PRMT5, WD45 and RIOK1 were also detected in caspase-8-co-IPs without stimulation. This suggests that these proteins may constitutively interact with procaspase-8. PRMT5 has been reported in some studies to bind non-specifically to beads, but this was not observed in these experiments. Indeed, no association of PRMT5 or WD45 and only a very weak signal for RIOK1 were obtained in control pulldowns, performed using the beads-only approach (Supplementary Fig. 2A, B). In addition, the signals corresponding to procaspase-8 were found in WD45-IP and PRMT5-co-IP (Supplementary Fig. 2C, D), further strengthening the evidence that procaspase-8 could be bound to the PRMT5 methylosome.

Taken together, the components of the PRMT5 methylosome complex were detected in caspase-8-co-IPs, which suggests the putative methylation of procaspase-8.

### In silico analysis predicted two putative arginine methylation sites on procaspase-8 that are highly conserved and often mutated in human cancers

Procaspase-8 is one of the DISC proteins that can be methylated by PRMT5. Indeed, the in silico analysis of putative PRMT5 methylation sites of procaspase-8 predicted with a high confidence two methylation sites in procaspase-8a at RG motifs: R233/G234 and R435/G436. For methylation site prediction, we used the PRmePRed web tool for identification of methylation sites at arginine residues [36].

**Fig. 2:**
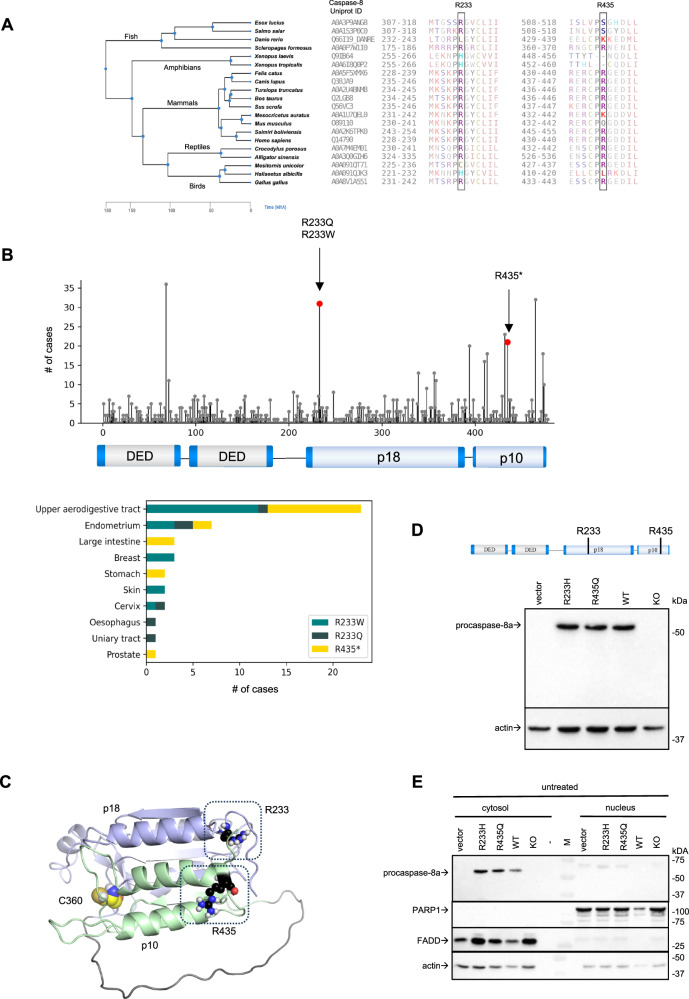
In silico analysis predicted two putative arginine methylation sites on procaspase-8 that are highly conserved and often mutated in human cancers. **A** Analysis of conservation of procaspase-8 R233 and R435 positions. The corresponding procaspase-8 protein regions for the representative higher vertebrate organisms are shown. The species tree and time points of the lineage diversification is presented on the left. The amino acid residues corresponding to human R233 and R435 residues are highlighted. **B** The prevalence of caspase-8 mutations in cancer was analysed using COSMIC database. All mutations mapped to their position in caspase-8a primary structure are shown in the upper panel. Three most prevalent mutations at R233 and R435 are shown in the various cancers in the lower panel: R233Q missense mutation is shown in black, R233W is shown in dark green and R435* nonsense mutation is shown in yellow. **C** The location of R233 and R435 in the three-dimensional structure of the C-terminal part of procaspase-8a is shown. The latter is computed using Alphafold [51]. The active site cysteine of caspase-8 is shown in yellow, while R233 and R435 are shown in blue colors. **D** Putative methylation residues are shown in procaspase-8a primary structure (upper panel). Procaspase-8a-R233H-pcDNA3 (R233H), procaspase-8a-R435Q-pcDNA3 (R435Q) as well as procaspase-8a-WT-pcDNA3 (WT) were transfected into HeLa-CD95-C8-KO cells (low panel). The transfection of pcDNA3 (vector) and non-transfected cells (KO) were used as negative controls. The protein expression was analysed by Western Blot. **E** HeLa-CD95-C8-KO cells were transfected as in (D). Cytosolic and nuclear fractions were prepared. The analysis of PARP1 and FADD was used as a fractionation control. The fractionation was analysed by Western Blot. One representative fractionation out of three independent experiments is shown.

Importantly, R233 and R435 were found to be highly conserved (Fig. 2A). The analysis of the evolutionary conservation of the procaspase-8 protein sequence revealed that both positions R233 and R435 in the human sequence are highly conserved in mammals confirming their putative functional role (Fig. 2A). Interestingly, in the case of R435, some species from avian and rodent taxonomic groups encode procaspase-8 with the amino acid changes corresponding to the R435Q substitution in the human sequence. In the case of R233, some organisms from the amphibian and bird species encode the R233H substitution. Thus, this analysis suggests the putative functional role of the R233 and R435 residues, which are common to higher vertebrates, and suggests the selection of amino acid mutations at these positions in the current study.

Furthermore, the analysis of cancer databases showed that the mutations at these two residues are quite common for caspase-8 in several human cancers (Fig. 2B). The R233 and R435 positions are frequently mutated leading to R233Q/R233W missense mutations and R435* nonsense mutation. This further supports the functional importance of these two arginine residues.

Both arginine residues are located in the C-terminal catalytic part of procaspase-8. R233 is located in the p18 large catalytic domain, whereas R435 is located in the p10 small catalytic domain (Fig. 2C, D). According to the available three-dimensional structures of active caspase-8, the two putative methylation sites are not located in close proximity to the active center of caspase-8, though their methylation might influence the conformational stability of the caspase-8 enzyme (Fig. 2C).

### Procaspase-8 mutations at the putative methylation sites inhibit CD95L-induced caspase activity and apoptosis

Procaspase-8a mutants R233H and R435Q (C8-R233H and C8-R435Q) were generated at predicted putative methylation sites. The selection of arginine substitutions was validated *in silico via* structural modeling to avoid perturbing the folding of procaspase-8. For this purpose, amino acid substitutions were selected that preserve protein energy as estimated by the Rosetta energy function [37]. In this way, both the R435Q and the R233H mutations were selected by us in the structural analysis in order to preserve protein function but to prevent methylation.

Procaspase-8a (C8-WT), C8-R233H and C8-R435Q were transiently transfected into HeLa-CD95-Crispr/Cas9 caspase-8^-/-^ (HeLa-CD95-C8-KO) cells (Fig. 2D). The concentration of the caspase-8 plasmids used for transfection was selected to achieve procaspase-8 expression slightly below the endogenous levels in parental HeLa-CD95 cells (Supplementary Fig. 3). This was done in order to avoid any effects due to the strong overexpression of this protein in the subsequent experiments.

**Fig. 3:**
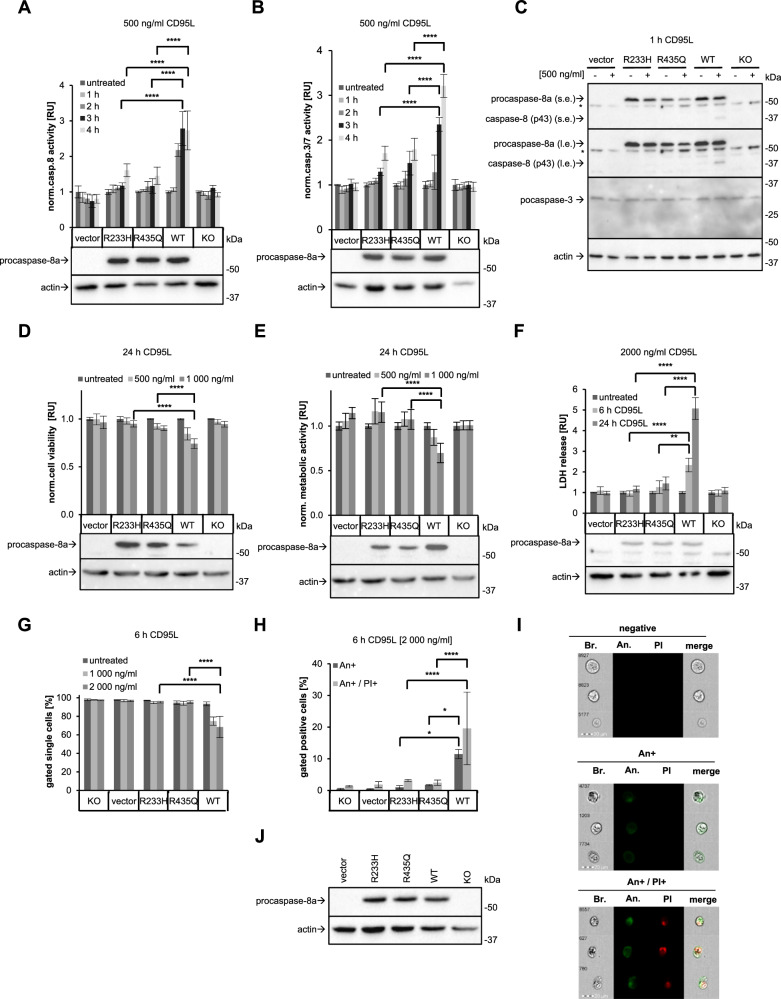
Procaspase-8 mutations at the putative methylation sites inhibit CD95L-induced caspase activity and apoptosis. **A**, **B** HeLa-CD95-C8-KO cells, which were transfected with pcDNA3 (vector), procaspase-8a-R233H-pcDNA3 (R233H), procaspase-8a-R435Q-pcDNA3 (R435Q) and procaspase-8a-WT-pcDNA3 (WT) or non-transfected (KO), were treated with the indicated amounts of CD95L for the indicated time intervals. Caspase-8 and -3 activities were determined by Caspase-Glo^®^8 Assay (**A**) and Caspase-Glo^®^3/7 Assay (**B**), respectively. Western Blot controls of transfection efficiency are shown in the lower part of the panels. **C** The HeLa-CD95-C8-KO cells were transfected as described before. This was followed by treatment with 500 ng/mL CD95L (+) for one hour and Western Blot analysis with the indicated antibodies were performed. One representative Western Blot out of three independent experiments is shown. **D**–**F** The HeLa-CD95-C8-KO cells were transfected as described before. Cells were treated with the indicated amounts of CD95L for indicated time intervals. Representative Western Blot analysis of transfection efficiency is shown in the lower part of the panels. **D** Cell viability was captured by measuring ATP levels using CellTiter-Glo^®^ 2.0 Cell Viability Assay. **E** Metabolic activity was captured by measuring metabolic level using RealTime-Glo™ MT cell Viability Assay. **F** LDH release was measured by LDH-Glo^®^ Cytotoxicity Assay. **G**, **H** HeLa-CD95-C8-KO cells were transfected as described above. After 24 hours, the cells were treated with indicated amounts of CD95L for 6 hours. Cells were stained by Annexin-V-FITC (An) and Propidium Iodide (PI) and then analysed by Imaging Flow Cytometry. **G** The amounts of viable cells (gated negative cells) after CD95L treatment are shown. **H** The amounts of An+ single-positive and An + /PI+ double positive cells after CD95L treatment are shown. **I** Representative images of viable (negative), An+ single-positive, An + /PI +  positive (double-positive) cells. **J** The representative Western Blot analysis of transfection efficiency is shown. **A**, **B**, **D**–**H** Mean and standard deviation are shown for three independent experiments. Statistical analysis was carried out by ordinary ONE-WAY ANOVA with subsequent Tukey-test (** significant; p < 0.01; *** significant; p < 0.001; **** significant; p < 0.0001). s.e. short exposure, l.e. long exposure, Bf bright field, An, Annexin V-FITC, PI Propidium Iodide.

The introduction of the R233H and R435Q mutations into procaspase-8a did not lead to changes in its expression or its cytosolic localisation (Fig. 2E), but resulted in the inhibitory effects on the CD95L-induced apoptotic signaling cascade. Indeed, CD95L-induced caspase-8- and caspase-3/7 activities, as measured by caspase-8 and -3/7 activity assays, respectively, were inhibited in HeLa-CD95-C8-R233H and HeLa-CD95-C8-R435Q cells as compared to HeLa-CD95-C8-WT cells (Fig. 3A, B; Supplementary Fig. 4A, B). Specifically, in these experiments, cells were stimulated with 500 ng/mL CD95L and caspase-8 and caspase-3/7 activities were monitored for one, two, three and four hours (Fig. 3A, B). In the case of HeLa-CD95-C8-R233H and HeLa-CD95-C8-R435Q cells, only a slight increase in caspase activity was observed compared to HeLa-CD95-C8-WT cells (Fig. 3A, B). It should be noted that the caspase-8 and caspase-3/7 activity assays are based on the cleavage of a short peptide-based substrate. However, these substrates are known to lack absolute specificity and to be cleaved not only by the above-mentioned caspases but also by the other caspases [38]. Therefore, these assays may actually indicate activation of the other caspases in addition to the one which activity is being tested, e.g., caspase-8 or caspase-3/7.

These results were consistent with the analysis of procaspase-8 and procaspase-3 processing by Western Blot in HeLa-CD95-C8-R233H and HeLa-CD95-C8-R435Q in comparison to HeLa-CD95-C8-WT cells (Fig. 3C, Supplementary Fig. 4C). The delayed rate of procaspase-8 and –3 cleavage was observed in HeLa-CD95-C8-R233H and HeLa-CD95-C8-R435Q compared to HeLa-CD95-C8-WT cells.

The decrease in caspase activity was consistent with the results of the analysis of the CD95L-induced loss of cell viability of HeLa-CD95-C8-R233H and HeLa-CD95-C8-R435Q compared to HeLa-CD95-C8-WT cells. The latter was monitored by assessing the total ATP levels and measuring cellular metabolism (Fig. 3D, E; Supplementary Fig. 4D). In these experiments, the introduction of the R233H and R435Q mutations into procaspase-8a resulted in an inhibitory effect on the CD95L-induced loss of cell viability. This was observed for both short-term and long-term CD95L treatment of HeLa-CD95-C8-R233H and HeLa-CD95-C8-R435Q cells by measuring total ATP levels and for the long-term treatment in the assays assessing cellular metabolism (Fig. 3D, E; Supplementary Fig. 4D).

The measurement of CD95L-induced LDH release also supported that the incorporation of the R233H and R435Q mutations into procaspase-8a inhibited CD95L-induced cell death in both short-term and long-term treatments, whereas the time-dependent increase in the CD95L-induced cytotoxicity was observed in HeLa-CD95-C8-WT cells (Fig. 3F). Finally, the analysis of the CD95L-induced apoptotic cell death using Annexin-V (An)/propidium iodide (PI) staining also showed that the introduction of the R233H and R435Q mutations into procaspase-8a blocked the CD95L-induced cell death (Fig. 3G–J, Supplementary Fig. 4E). Taken together, these experiments have demonstrated that the introduction of mutations at putative procaspase-8a methylation sites R233 and R435 leads to the inhibitory effects on CD95L-induced apoptosis.

### Procaspase-8 may undergo symmetric di-methylation

**Fig. 4:**
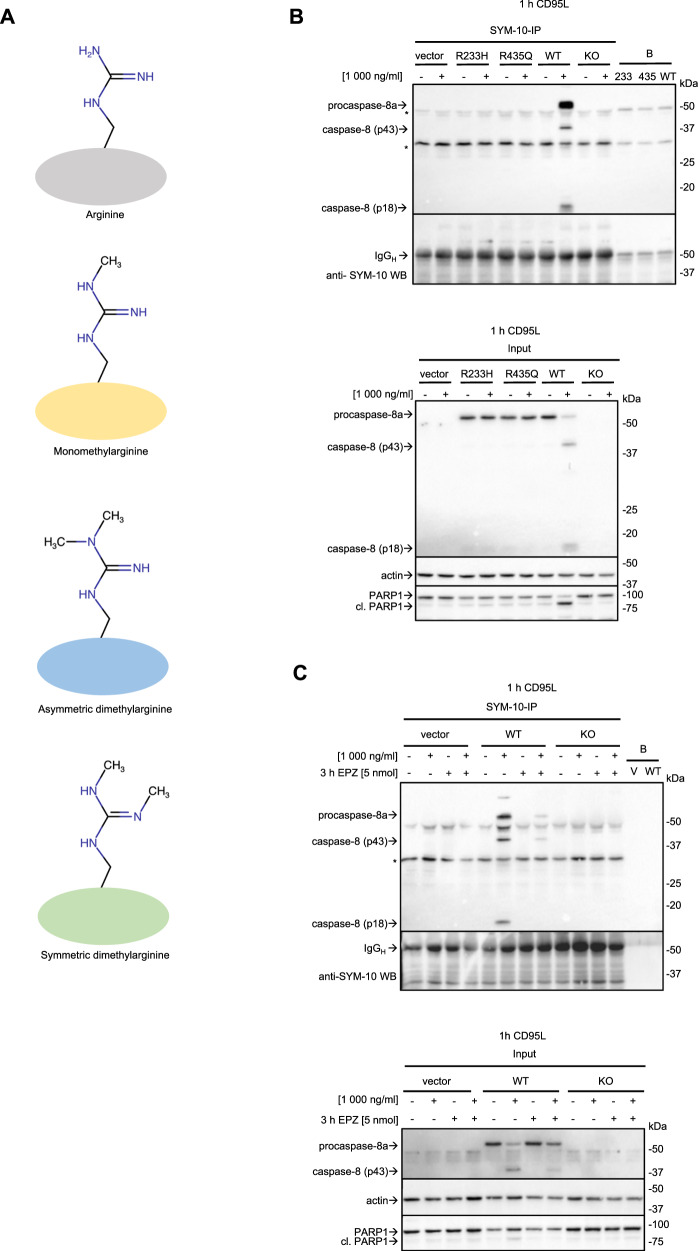
Procaspase-8 may undergo symmetric di-methylation. **A** Three types of arginine methylation are shown schematically. **B** HeLa-CD95-C8-KO cells transfected with pcDNA3 (vector), procaspase-8a-R233H-pcDNA3 (R233H), procaspase-8a-R435Q-pcDNA3 (R435Q) and procaspase-8a-WT-pcDNA3 (WT) as well as non-transfected (KO) were treated with 1000 ng/mL of CD95L (+) for one hour (h). This was followed by SYM-10-IP with subsequent Western Blot analysis. In these experiments, immunoprecipitation has been carried out without immobilization, which we designate as IP. Lysates (Input), IPs and bead-only control (B) are shown. One representative Western Blot analysis out of three independent experiments is shown. **C** HeLa-CD95-C8-KO cells transfected with pcDNA3 (vector), and procaspase-8a-WT-pcDNA3 (WT) as well as non-transfected (KO) were treated with 1000 ng/mL of CD95L (+) for one hour in the presence or absence of EPZ inhibitor. This was followed by SYM-10-IP with subsequent Western Blot analysis. Lysates (Input), IPs and bead-only control (**B**) are shown. One representative Western Blot analysis out of three independent experiments is shown. IP immunoprecipitation, B beads-only pulldown, s.e. short exposure, l.e. long exposure, v vector. **B**, **C** Non-specific signals are marked with *. The signal for the heavy chain of antibody used for IP is marked with IgG_H_.

PRMT5 is able to methylate its substrates in two ways, *via* mono-methylation and symmetric di-methylation [26] (Fig. 4A). To explore whether procaspase-8 undergoes symmetric di-methylation we have performed IPs with the antibodies that recognize symmetric di-methylated proteins (SYM-10 antibodies). The SYM-10-IP should pulldown the proteins that undergo symmetric di-methylation. This IP was carried out from CD95L-stimulated HeLa-CD95-C8-KO cells transfected with procaspase-8a-WT, R233H, R435Q or vector control, e.g. HeLa-CD95-C8-WT, HeLa-CD95-C8-R233H, HeLa-CD95-C8-R435Q, or HeLa-CD95-C8-KO cells, respectively (Fig. 4B). The Western Blot analysis of SYM-10-IPs has revealed the presence of procaspase-8a-WT in SYM-10-IPs upon CD95L stimulation. However, no procaspase-8a-R233H or procaspase-8a-R435Q was detected in SYM-10-IPs (Fig. 4B). Furthermore, the p43 and p18 cleavage products of procaspase-8a were detected in the SYM-10-IP indicating that these two cleavage products can also undergo symmetric di-methylation. These results suggest a potential symmetric di-methylation of procaspase-8.

EPZ (EPZ015666) is a specific inhibitor of PRMT5. Next, HeLa-CD95-C8-WT cells were co-treated with CD95L and EPZ. The SYM-10-IP from these cells showed that the addition of the EPZ inhibitor reduced the levels of procaspase-8a and its cleavage products p43 and p18 in the SYM-10-IP (Fig. 4C). This indicates a decrease in di-methylated procaspase-8 upon administration of EPZ, supporting the role of PRMT5 in the methylation of procaspase-8.

**Fig. 5:**
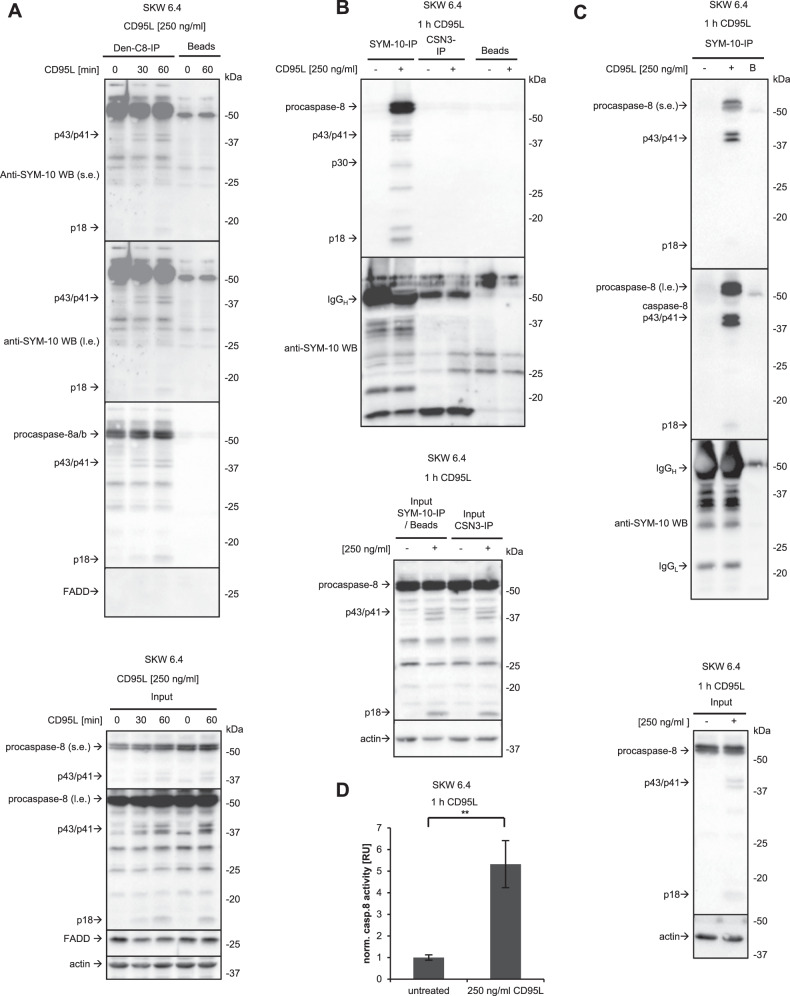
Caspase-8 activity is detected in SYM-10-IP. **A**, **B** SKW 6.4 cells were treated with the indicated concentrations of CD95L for the indicated time intervals. **A** Denaturing (Den) Caspase-8-IP (Den-C8-IP) was analysed with anti-SYM-10 and anti-caspase-8 Western Blot. The position of p43/p41 and p18 bands detected with SYM-10 antibodies are indicated by arrows. **B** SYM-10-IP has been carried out. IPs and Lysate control (Input) were analysed by Western Blot with indicated antibodies. CSN3-IP was used as isotype control IP. One representative experiment out of three is shown. Heavy chain of antibody is marked with IgG_H_**. C** SYM-10-IP has been carried out. IPs and Lysate control (Input) were analysed by Western Blot with indicated antibodies. One representative experiment out of three is shown. **D** Caspase-8 activity at SYM-10-IPs shown in (**C**) was determined using the IPs. Mean and standard deviation are shown for three independent experiments. Statistical analysis was carried out by unpaired student t-test (** significant; p < 0.01). IP immunoprecipitation, B beads-only pulldown, s.e. short exposure, l.e. long exposure, IgG_H_ Heavy chain of immunoprecipitating antibody, IgG_L_ light chain of immunoprecipitating antibody.

To provide further evidence for symmetric di-methylation, procaspase-8-IP from SKW 6.4 cells was performed with the addition of SDS and heating of the lysates to 95 °C, *i.e*. a so-called ‘denaturing’ IP of procaspase-8 (Den-C8-IP). The addition of SDS to Den-C8-IP led to the immunoprecipitation of procaspase-8 alone without any core DISC components such as FADD (Fig. 5A). Moreover, the Den-C8-IPs with subsequent Western Blot analysis using SYM-10 antibodies have provided further evidence for the possible symmetric di-methylation of procaspase-8. Indeed, the bands of the size of the caspase-8 cleavage products p43/p41 and p18 were detected in the Den-C8-IP from CD95L-stimulated SKW 6.4 cells using anti-SYM -10 Western Blot (Fig. 5A). As highlighted above, IPs performed under denaturing conditions contain only the bait, which in this experiment will be caspase-8. Naturally, PTM-modified caspase-8 isoforms will also be present in this pulldown. This gives further support for the symmetric di-methylation of procaspase-8 since anti-SYM-10 Western Blot analysis revealed the putative caspase-8 signals in the Den-C8-IPs.

Since PRMT5 methylates its substrates *via* mono-methylation and symmetric di-methylation, we also investigated if procaspase-8 might undergo mono-methylation. However, the analysis of Den-C8-IP by Western Blot with anti-mono-methylation (MMA) antibodies did not reveal any signals corresponding to the molecular weight of procaspase-8 or its cleavage products (Supplementary Fig. 5A). This observation was further supported with the IP from SKW 6.4 cells without the addition of SDS using anti-MMA antibodies. This also did not result in immunoprecipitation of procaspase-8 (Supplementary Fig. 5B). Taken together, these experiments did not reveal any mono-methylation of procaspase-8.

### Pharmacological inhibition of methylation reduces CD95L-induced caspase activity

It should be noted that in HeLa-CD95-C8-KO cells transfected with procaspase-8a-WT, procaspase-8a cleavage upon CD95L stimulation, was strongly decreased upon administration of EPZ (Fig. 4B). In line with the decrease in procaspase-8a processing, the CD95L-induced cleavage of PARP1, was also downmodulated upon administration of EPZ (Fig. 4B). These results strongly suggest that the catalytic activity of caspase-8 and effector caspases is inhibited upon pharmacological inhibition of PRMT5.

To further investigate the link between caspase-8 activity and symmetric di-methylation, SYM-10-IPs were carried out from CD95L-stimulated and non-stimulated SKW 6.4 cells with the following analysis of caspase-8 activity. SYM-10-IPs from CD95L-stimulated cells contained the procaspase-8 cleavage products p43/p41 and p18 (Fig. 5B, C). Furthermore, caspase-8 activity can be detected in these IPs upon CD95L stimulation (Fig. 5D). The latter provides further support that symmetrically di-methylated caspase-8 has a catalytic activity.

**Fig. 6:**
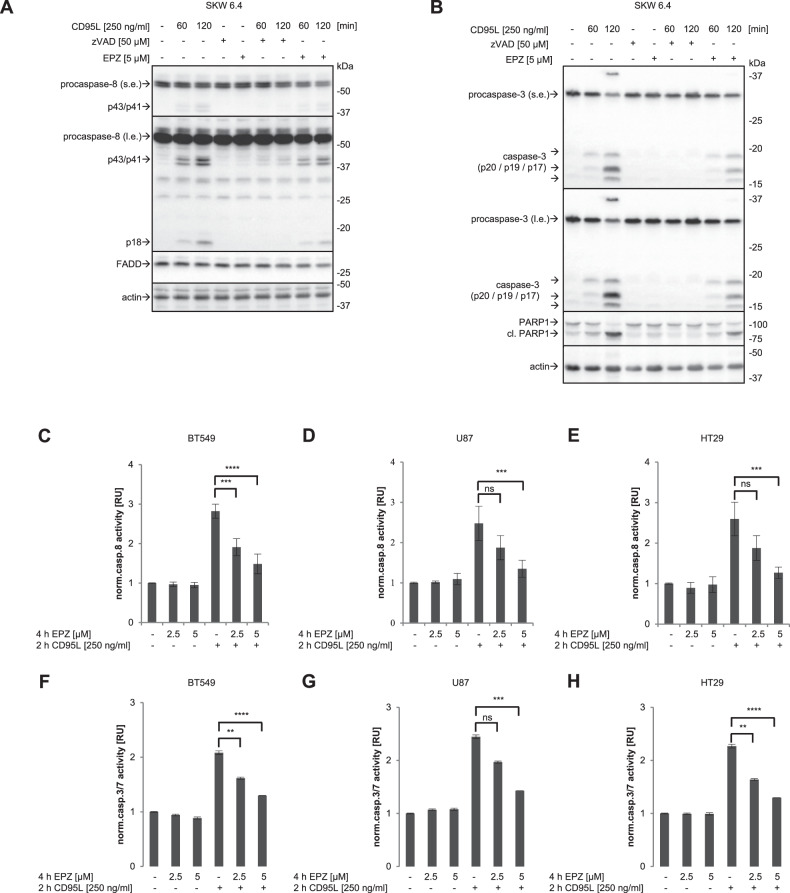
Pharmacological inhibition of PRMT5 blocks CD95L-induced caspase cascade. **A**, **B** SKW 6.4 cells were treated with 250 ng/mL of CD95L, zVAD-fmk and EPZ for the indicated time intervals with subsequent Western Blot analysis. One representative experiment out of three independent ones is shown. **C**–**H** BT549 cells (**C**, **F**), U67 (**D**, **G**) and HT29 (**E**, **H**) were treated with CD95L for two hours (**C**–**E**) or four hours (**F**–**H**). Caspase-8 and -3/7 activities were determined by Caspase-Glo^®^8 Assay (**C**–**E**) and Caspase-Glo^®^3/7 Assay (**F**–**H**), respectively. **A**–**H** EPZ pre-treatment was performed for two hours before CD95L treatment and zVAD-fmk pre-treatment was performed for one hour before CD95L stimulation. Statistical analysis was carried out by ordinary ONE-WAY ANOVA with Tukey-test (ns not significant; *p* > 0.05, * significant; *p* < 0.05, ** significant; p < 0.01; *** significant; p < 0.001; **** significant; p < 0.0001). s.e. short exposure.

Consistent with the results obtained in HeLa-CD95 cells (Fig. 4B), the rate of procaspase-8a/b processing in CD95L-treated SKW 6.4 cells was also decreased upon EPZ administration, as evidenced by the diminished amounts of p43/p41 in the total cellular lysates (Fig. 6A). This analysis further indicates that the inhibition of methylation by means of the addition of EPZ decreases the rate of procaspase-8 activation.

In line with the decrease of procaspase-8 activity, the analysis of caspase-3 and PARP1 cleavage by Western Blot in CD95L-treated SKW 6.4 cells showed that EPZ delayed the cleavage of the effector caspase-3 as well as its substrate PARP1 (Fig. 6B). These effects were consistent with the inhibitory effects of AMI-5, which is the pan-methylation inhibitor of protein arginine methyltransferases. In particular, AMI-5 inhibited the activity of effector caspases-3 and -7 in SKW 6.4 cells (Supplementary Fig. 6A). Moreover, administration of EPZ in breast carcinoma BT549, glioblastoma U87 and colon carcinoma HT29 cells led to the decrease in CD95L-induced caspase-8 (Fig. 6C–E) and caspase-3/7 activities (Fig. 6F–H).

Collectively, these results show that the pharmacological inhibition of PRMT5 leads to the inhibitory effects on the caspase cascade.

### Pharmacological inhibition of methylation decreases CD95L-mediated apoptosis upon short-term stimulation

**Fig. 7:**
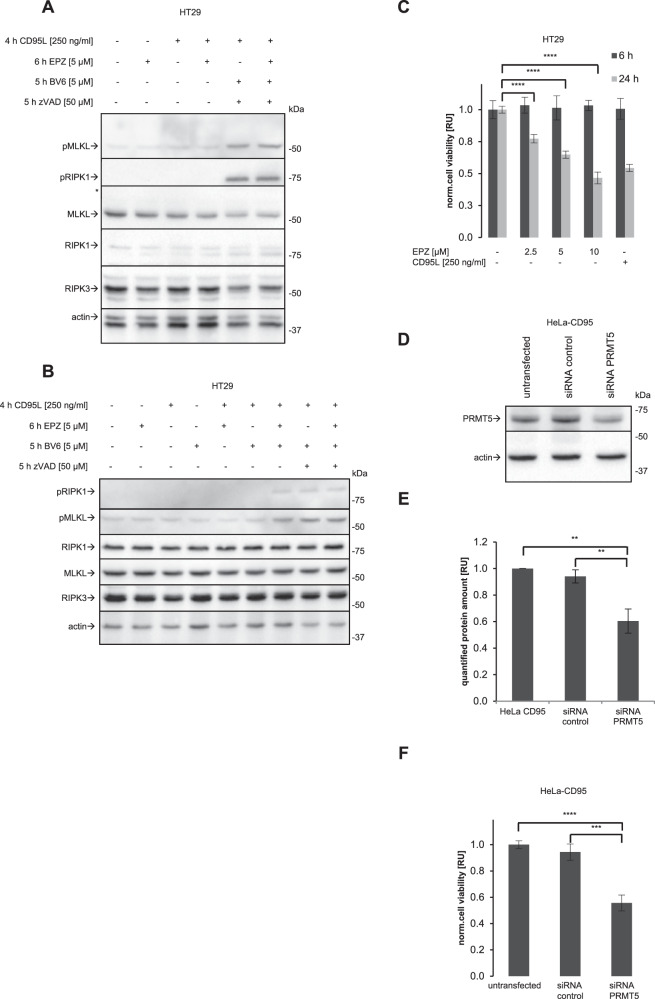
Pharmacological and genetic downmodulation of PRMT5 activity reduces cell viability upon long-term treatment. **A**, **B** HT29 cells were treated with the indicated concentrations of CD95L, EPZ, zVAD-fmk and BV6 for indicated time intervals. Necroptosis markers were evaluated by Western Blot with the indicated antibodies. **C** HT29 cells were treated with the indicated concentrations of EPZ and CD95L for the indicated time intervals. Cell viability was captured by measuring ATP levels using CellTiter-Glo^®^ 2.0 Cell Viability Assay. **C**–**F** HeLa CD95 cells were transfected by siRNA of PRMT5 and control siRNA. Western Blot analysis (**D**) and Quantification (**E**) of transfection efficiency are shown. **F** Effects of the PRMT5 KD on cell viability was captured by measuring ATP levels using CellTiter-Glo^®^ 2.0 Cell Viability Assay and normalized against untransfected cells. **C**, **E**, **F** Mean and standard deviation are shown for three independent experiments. Statistical analysis was carried out by ordinary ONE-WAY ANOVA and followed Tukey-test (** significant; p < 0.01; *** significant; p < 0.001; **** significant; p < 0.0001).

The next step was to investigate the impact of pharmacological inhibition of methylation on extrinsic cell death pathways. Since caspase-8 is a negative regulator of necroptosis, we next examined whether administration of EPZ could affect CD95L-mediated necroptosis induction, which was induced by co-treatment of CD95L, IAP inhibitor BV6 and zVAD-fmk in HT29 cells. Western Blot analysis showed phosphorylation of MLKL and RIPK1 upon CD95L/BV6/zVAD-fmk treatment, but no effect on this phosphorylation was detected upon co-administration of EPZ, e.g. upon CD95L/BV6/zVAD-fmk/EPZ compared to CD95L/BV6/zVAD-fmk treatment (Fig. 7A). The latter may be related to the fact that EPZ inhibits caspase-8 activity, which is already inhibited by zVAD-fmk administration in these experiments. Importantly, however, treatment with CD95L, EPZ and BV6 alone, without the addition of zVAD-fmk, induced the phosphorylation of MLKL and RIPK1, supporting the hypothesis that EPZ inhibits caspase-8 activity and thereby may also promote the induction of necroptosis (Fig. 7B).

Next, we aimed to analyse the effect of inhibiting caspase-8 methylation on long-term co-treatment with CD95L. To this end, we first tested the viability of the cells upon EPZ treatment alone (Fig. 7C). However, this analysis revealed that long-term co-treatment with EPZ alone severely reduced cell viability, precluding further analysis of the effects of pharmacological inhibition of PRMT5 on caspase-8 activity upon long-term CD95L/EPZ stimulation. This is consistent with previous reports on the essential role of PRMT5 in key cellular processes [39]. In line with these results, siRNA silencing of PRMT5 also resulted in a significant loss of cell viability as early as 24 hours after transfection (Fig. 7D–F, Supplementary Fig. 6B, C). These results precluded further investigation of the effects of genetic or pharmacological inhibition of PRMT5 on CD95L-mediated cell death upon long-term treatment.

**Fig. 8:**
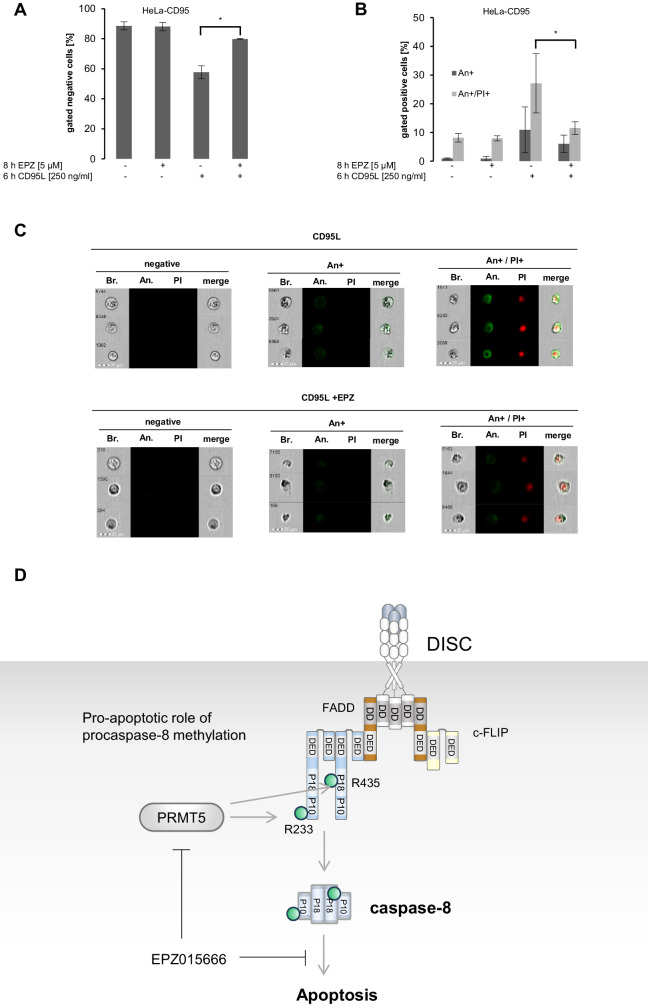
Pharmacological inhibition of PRMT5 blocks CD95L-induced apoptosis. **A**–**C** HeLa-CD95 cells were treated with indicated amounts of CD95L for 6 hours. Cells were stained by Annexin-V-FITC (An) and Propidium Iodide (PI) and then analysed by Imaging Flow Cytometry. **A** The amounts of viable cells (negative cells) after CD95L treatment are shown. **B** The amounts of An+ single-positive and An + /PI+ double-positive cells after CD95L treatment are shown. **C** Representative images of viable (negative), An + single-positive, An + /PI +  positive (double-positive) cells. **A**, **B** Statistical analysis was carried out by ordinary ONE-WAY ANOVA with Tukey-test (ns not significant; *p* > 0.05, * significant; *p* < 0.05). Bf bright field, An Annexin V-FITC, PI Propidium Iodide. **D** The proposed role of caspase-8 methylation in the CD95 apoptotic cascade. The assembly of the CD95 DISC consisting of CD95, FADD, procaspase-8 and c-FLIP is shown leading to caspase-8 activation. The putative caspase-8 methylation sites, arginines 233 and 435, are shown as green spheres. The proposed effects of PRMT5 on caspase-8 activation are shown schematically.

Despite the effects observed upon long-term treatment, we analysed the effects of EPZ on CD95L-mediated cell death upon short-term stimulation, considering that under these conditions, PRMT5 inhibition does not yet affect cell viability. Indeed, when HeLa-CD95 cells were stimulated with 250 ng/ml CD95L for 6 hours, the addition of EPZ blocked the rate of cell death as measured by imaging flow cytometry (Fig. 8A–C). The latter results are consistent with the data obtained above and show that inhibition of PRMT5 upon short-term treatment leads to the inhibitory effects on caspase-8 activation, caspase cascade and subsequent apoptosis.

## Discussion

Activation of procaspase-8 at the CD95 DISC plays a key role in the control of apoptotic and anti-apoptotic pathways [4, 17, 40–43]. The important role in this intricate control belongs to PTMs of procaspase-8 and their role is only starting to be deciphered [4]. In this study, *via* mass spectrometry screening, pharmacological inhibition and biochemical analysis it was demonstrated that procaspase-8 can be targeted by the PRMT5/RIOK1/WD45 methylosome complex. Furthermore, the possible pro-apoptotic role of procaspase-8 methylation was proposed, which suggests the unexpected regulatory methylation network of life and death decisions in a cell.

The PRMT5 methylosome complex, which includes PRMT5, RIOK1 and WD45, can be localised in the cytosol, which was confirmed in this study [29]. The cytosolic localisation of the PRMT5/RIOK1/WD45 methylosome would be in accordance with the methylation of the cytosolic targets such as procaspase-8. Besides the cytosol, procaspase-8 has also been reported to be found in the nucleus, where it plays a key role in several non-apoptotic processes [42, 44]. However, in our study, we investigated the apoptotic role of procaspase-8 methylation and did not consider procaspase-8 involvement in the non-apoptotic pathways, which take place in the nucleus. This research can be done in the future.

The epigenetic control of gene expression mediated *via* methylation attracts high attention in contemporary cancer research. In this study, we have considered the effects of methylation at the protein level focusing on the key initiator caspase of extrinsic apoptosis, procaspase-8. Methylation at the protein level adds complexity to the understanding of the cellular methylome in the cell fate and shows that the methylation of core apoptotic regulators might provide the additional sensitive switches in the regulation of life/death decisions.

We can show that procaspase-8 can be immunoprecipitated *via* anti-SYM-10 antibodies, which recognize only symmetric di-methylation. This indicates that procaspase-8 can undergo symmetric di-methylation. Interestingly, we can only detect procaspase-8 in the SYM-10-IPs upon CD95L stimulation. Surprisingly, we also have detected the core components of the PRMT5 complex co-immunoprecipitated with procaspase-8 as well as CD95 in the absence of CD95L stimulation. The possibility of CD95 methylation by PRMT5 has to be investigated in the future. For procaspase-8 methylation, the results of the SYM-10-IPs suggest that procaspase-8 methylation may be induced or greatly increased upon CD95L stimulation, as indicated by the detection of procaspase-8a/b and its cleavage products in the SYM-10-IPs from CD95L-stimulated cells only. The latter were not observed in SYM-10-IP without CD95L stimulation. The methylation upon CD95L stimulation might be due to the conformational changes of procaspase-8 upon its binding to the DISC that likely makes arginine residues more accessible for the action of methylation enzymes.

All members of the PRMT protein family add methyl groups to arginine residues. The PRMT family members are divided into two types: Type 1 includes PRMT1, 2, 3, 4, 6 and 8, which might induce mono-methylation and asymmetric di-methylation. PRMT 5, 7 and 9 belong to the type 2 family members. This family of enzymes induces mono-methylation and symmetric di-methylation [29, 45]. In our study, we focused primarily on the PRMT5 methylosome complex since PRMT5, RIOK1 and WD45 were detected in the mass spectrometry screening with the higher scores compared to the other components of the human methylome. Furthermore, our data indicate that procaspase-8 undergoes symmetric di-methylation, which is in accordance with the described specificity of PRMT5. However, we do not exclude that the other PRMTs can act on various components of the extrinsic cell death network by inducing mono- and di-methylation on arginine residues. In this context, it should be mentioned that arginine methylation of RIPK3 by PRMT5 has recently been described, and that methylation of c-FLIP_L_ by PRMT5 and PRMT1 has been reported to be important for c-FLIP_L_ degradation [46, 47]. The further role of arginine methylation in extrinsic cell death pathways needs to be addressed in future studies.

Furthermore, bioinformatic analysis shows the presence of two caspase-8 methylation sites in the p10 and p18 subunits at RG consensus motifs: R233/G234 and R435/R436. The latter would be in accordance with the detection of procaspase-8a/b and its cleavage products p43/p41 and p18 in SYM-10-IP as symmetrically di-methylated proteins. Importantly, these two arginine residues are highly conserved and mutations at these two residues are common in several cancers (Fig. 2A, B). This further demonstrates the important role of these two arginine residues for caspase-8 activity and apoptosis.

Two RG sites are located within the catalytic domains of procaspase-8. R233 resides at p18 large catalytic domain, while R435 resides at p10 small catalytic domain (Fig. 2C). Both sites are not situated in close structural proximity to the active center of caspase-8 and cannot directly control its activity *via* putative interactions. This suggests that the methylation at these two sites might be essential for the conformational stability of the caspase-8 enzyme thereby controlling its catalytic activity (Fig. 8D). Indeed, the introduction of mutations at these two sites strongly blocked CD95L-induced procaspase-8 activity as well as cell death in the HeLa-CD95-C8-KO cells transfected with the respective procaspase-8 mutants. The cell death measurements were performed with short-term and long-term treatments. Further, the pharmacological inhibition of PRMT5 also shown inhibitory effects on caspase-8 activity and apoptosis, while having promoting effects on activation of necroptosis markers, which were measured only upon short-term treatments. It has to be noted that the impact of pharmacological inhibition of caspase-8 arginine methylation on cell death upon long-term treatment could not be investigated due to the general effects of PRMT5 inhibitors on the cellular methylation networks leading to the loss of cell viability, as reported by others [39]. Similar effects were also found in this study by examining the long-term effects of EPZ treatment or PRMT5 downregulation. Taken together, the introduction of mutations and pharmacological inhibition indicates the pro-apoptotic effects of the methylation of procaspase-8. The latter might be related to the influence of arginine methylation on the conformational stability of the caspase-8. In the future pharmacological studies it might be considered to create the small molecules that would promote methylation state of these two arginines at caspase-8 and thereby increase apoptosis.

It has to be mentioned that PRMT5 is considered as one of the promising targets in cancer due to its effects on the cell proliferation, invasion and migration [48]. Furthermore, PRMT5 has been shown to be upregulated in several cancers and genetic or pharmacological downmodulation of its activity promotes cell death of cancer cells [39, 48]. However, the results obtained in this study suggest that PRMT5 can also promote apoptosis *via* methylation of caspase-8, which may also be important for breaking the resistance of cancer cells and must be taken into account in the design of therapies targeting the methylation networks of the cell.

In summary, this study suggests that procaspase-8 undergoes symmetric di-methylation, which appears to have a pro-apoptotic effect. This adds yet another checkpoint to the caspase-8 activation mechanism and opens a new perspective for controlling the extrinsic apoptosis network.

## Material & methods

### Antibodies and reagents

All chemicals were of analytical grade and purchased from AppliChem (Darmstadt, Germany), CarlRoth (Karlsruhe, Germany), Gibco™ (Carlsbad, USA), Merck (Darmstadt, Germany) or SigmaAldrich (Taufkirchen, Germany). Recombinant CD95L was produced as described in [49]. zVAD-fmk (N-1510, Bachem) Enzo Life Sciences), EPZ / EPZ015666 (Selleck Chemicals GmbH; Planeck, Germany); AMI-5 (539211-50MG, Merck (Darmstadt, Germany)) and BV6 (#OR-502922, Genentech Inc. (California, USA)) were added to cells in indicated concentrations. The following antibodies were used for Western Blot analysis: polyclonal anti-CSN3 antibody (ab111406); polyclonal anti-RIP3 antibody (ab226297) from Abcam (UK) monoclonal anti-pMLKL antibody (ab184718), polyclonal anti-RIOK1 antibody (#NBP1-30104) from Novus Biologicals (Littleton, USA); polyclonal anti-symmetric di-methylation (SYM-10) antibody (#07-412) from Merck (Darmstadt, Germany); polyclonal anti-caspase-3 antibody (#9662), polyclonal anti-Endonuclease G (Endo G) antibody (#4969), polyclonal anti-MEP50 (WD45) antibody (#2823), monoclonal anti-MLKL antibody (#14993); polyclonal anti-Mono-Methyl Arginine (MMA) antibody (#8711), polyclonal anti-PARP1 antibody (#9542), monoclonal anti-Parkin (Prk8) antibody (#4211), monoclonal anti-pRIP1 antibody (#65746); polyclonal anti-PRMT5/Skb1Hs antibody (#2252) and monoclonal anti-RIP (XP) antibody (#3493) from Cell Signaling Technology (USA); polyclonal anti-GAPDH antibody (sc-48166) and polyclonal anti-CD95 antibody (sc-715) from Santa Cruz, (USA); polyclonal anti-actin antibody (A2103) from Sigma-Aldrich, (Germany). Monoclonal anti-caspase-8, anti-FADD and anti-APO-1 antibodies were kindly provided by Prof. P. H. Krammer, DKFZ, Heidelberg. Horseradish peroxidase (HRP)-conjugated goat anti-mouse IgG1, IgG2b, goat anti-rabbit and rabbit anti-goat were from Southern Biotech (Alabama, USA).

### Cell culture

Human cervical cancer HeLa-CD95-C8-KO cells were generated from HeLa cells with CD95 overexpression (HeLa-CD95) [35]. The cells were maintained in DMEM/Hams F12 Media (PAN-Biotech GmbH), supplemented with 10% heat-inactivated fetal calf serum, 1% Penicillin-Streptomycin and 0.0001% Puromycin in 5% CO_2_. Human breast adenocarcinoma cells BT549 and Human B lymphoma SKW 6.4 cells [9] were maintained in RPMI 1640 (Thermo Fisher Scientific Inc., USA), supplemented with 10% heat-inactivated fetal calf serum and 1% Penicillin-Streptomycin in 5% CO_2_. Human glioblastoma cells U87 and colon cancer cells HT29 were maintained in DMEM Media (Gibco™, USA), supplemented with 10% heat-inactivated fetal calf serum and 1% Penicillin-Streptomycin in 5% CO_2._

### Structural modeling

Protein energy changes upon introduction of point mutations into procaspase-8 were estimated using the RosettaDDGPrediction method implemented within PyRosetta framework [37]. The structure of procaspase-8 was derived from the AlphaFold database [50, 51].

### Analysis of conservation of procaspase-8 R233 and R435 positions

Analysis of conservation was carried out using the following programmes: the species tree was constructed by the TimeTree web server [52]. Sequence alignment of procaspase-8 sequences was carried out using MAFFT software [53]. Representative caspase-8 sequences of different species were derived from the UniProt database [54] .

### Mutagenesis of caspase-8

The mutagenesis of caspase-8a-pcDNA3 was performed by Genscript, USA. Caspase-8a-R233H-pcDNA3 (caspase-8a-R233H) and caspase-8a-R435Q-pcDNA3 (caspase-8a-R435Q) were generated from procaspase-8a-pcDNA3 (# 11817, Addgene, USA) (caspase-8a-WT).

### Caspase-8 transfections

1 * 10^6^ HeLa-CD95-C8-KO cells were seeded in 10 mL media into each well of a 10 cm-plate one day before transfection. Just before the transfection, the old media was aspirated and 8 mL of fresh media (antibiotic-free) was added to the cells. 2 µg of empty vector (pcDNA3), caspase-8a-R233H, caspase-8a-R435Q or caspase-8a-WT were mixed separately with 250 µL OPTIMEM (Gibco™, USA). Each sample was mixed with 24 µL DreamFect Gold (ORBIOSCIENCES, USA) in 250 µL OPTIMEM and incubated for 20 min at room temperature. After 20 min incubation, the samples were added to the cells. After two hours of incubation at 37 °C and 5% CO_2_, 100 µM zVAD-fmk was added to each well. Transfections on 6-well plates are performed with a lower dose, to get to the same amount of plasmid per 1*10^6^ cells: just before the transfection, the old media was aspirated and 2 mL of fresh media (antibiotic-free) was added to the cells. 0.5 µg of empty vector (pcDNA3), caspase-8a-R233H, caspase-8a-R435Q or caspase-8a-WT were mixed separately with 100 µL OPTIMEM (Gibco™, USA). Each sample was mixed with 4 µL DreamFect Gold (ORBIOSCIENCES, USA) in 100 µL OPTIMEM and incubated for 20 min at room temperature. After 20 min of incubation, the samples were added to the cells. After two hours of incubation at 37 °C and 5% CO_2_, 100 µM zVAD-fmk was added to each well. All experiments were performed after 22 h under standard conditions.

### PRMT5 knock down

For the transfection, FlexiTube siRNA technology from QIAGEN (Hilden, Germany) was used following the customers advice. PRMT5 *knockdown* (KD) was done by PRMT5 siRNA Premix (FlexiTube siRNA Premix (#1027420)) and as control siRNA of Cdk4_5 (#1027417) was used. 0.5 * 10^6^ HeLa-CD95 cells were seeded in 2 mL media into each well of a 6-well-plate or 1.2 * 10^4^ HeLa-CD95 cells were seeded in 100 µL media into each well of a 96-well-plate were seeded one day before transfection. Just before the transfection, the old media was aspirated and 2 mL (6-well plate) or 100 µL (96-well plate) of fresh media (antibiotic-free) was added to the cells. Transfection was performed by adding the FlexiTube siRNA following the customer’s protocol. 25 nM of siRNA was used for the PRMT5-KD and siRNA control. Cells were incubated for 24 h under standard conditions, which was followed by the experiments.

### Cell viability measurements by ATP and metabolic assays

1.2 * 10^4^ of HeLa-CD95-C8-KO cells after transfection with procaspase-8a constructs (one day before treatment) or 2 * 10^4^ of SKW 6.4 cells were seeded into each well in 96-well plates. Cells were stimulated in a volume of 50 µL. For ATP assay, following the manufacturer’s instructions (CellTiter-Glo^®^ 2.0 Cell Viability Assay, Promega, Germany), measurements were performed by addition of 50 µL CellTiter-Glo^®^ solution to each well. The luminescence intensity was analysed in duplicates using the microplate reader Infinite M200pro (Tecan, Switzerland). The values were normalized to the viability of untreated cells. One relative unit (RU) corresponds to the viability of untreated cells.

For metabolic assay, following the manufacturer’s instructions (RealTime-Glo™ MT cell Viability Assay, Promega Germany), measurements were performed *via* addition of 50 µL metabolic Substrate (2% MT cell viability substrate and 2% NanoLuc™Enzyme) to each well, directly before stimulation. The luminescence intensity was analysed in duplicates using microplate reader Infinite M200pro (Tecan, Switzerland). The values were normalized to the viability of untreated cells. One relative unit (RU) corresponds to the viability of untreated cells.

### Cytotoxicity measurements by lactatedehydrogenase (LDH) assay

1.2 * 10^4^ HeLa-CD95-C8-KO cells after transfection with procaspase-8a constructs were seeded into each well of 96-well plates one day prior to treatment. Cells were stimulated in a volume of 50 µL medium. LDH assay was performed following the manufacturer’s instructions (LDH-Glo^®^ Cytotoxicity Assay, Promega, Walldorf, Germany). In brief, 2 µL cell supernatant was added to 198 µL LDH storage buffer (200 mM Tris HCl, pH 7.3, 10% glycerol, 1% BSA). Then, 50 µL of this solution was mixed with 50 µL LDH-Substrate. The luminescence signal was analysed in duplicates using microplate reader Infinite M200pro (Tecan, Switzerland). The values were normalized to the LDH release of untreated cells. One relative unit (RU) corresponds to the LDH release of untreated cells.

### Caspase-3/7 activity assay

1.2 * 10^4^ of HeLa-CD95-C8-KO cells after transfection with procaspase-8a constructs (1 day before treatment), 1.2 * 10^4^ of BT549, HT29 or U87 or 2*10^4^ of SKW 6.4 cells were seeded into each well in 96-well plates. Cells were stimulated in a volume of 50 µL. Following the manufacturer’s instructions (Caspase-Glo^®^3/7 Assay, Promega, Germany), measurements were performed *via* addition of 50 µL of the Caspase-Glo^®^3/7 solution to each well. The luminescence intensity was analysed in duplicates using the microplate reader Infinite M200pro (Tecan, Switzerland). The values were normalized to the signal of untreated cells. One relative unit (RU) corresponds to the activity of untreated cells.

### Caspase-8 activity assay

1.2 * 10^4^ of HeLa-CD95-C8-KO cells after transfection with procaspase-8a constructs (1 day before treatment), 1.2 * 10^4^ of BT549, HT29 or U87 or 2 * 10^4^ of SKW 6.4 cells were seeded into each well in 96-well plates. Cells were stimulated with the indicated treatments in a volume of 50 µL. After the indicated time of incubation at 37 °C and 5% CO_2_, samples were measured according to the manufacturer’s instructions (Caspase-Glo^®^8 Assay, Promega, Germany) by addition of 50 µL Caspase-Glo^®^8 solution to each well. 0.3% of MG-132-Inhibitor was used in these experiments. The luminescence intensity was analysed in duplicates by the microplate reader Infinite M200pro (Tecan, Switzerland). The values were normalized to the caspase-8 activity of untreated cells. One relative unit (RU) corresponds to the caspase-8 activity of untreated cells.

### Cell death measurements by imaging flow cytometry

Analysis of cell death induction was performed with FlowSight® Imaging Flow Cytometer (Amnis/MerckMillipore, USA). HeLa-CD95 cells were treated with indicated concentration CD95L. Samples were stained with Annexin V-FITC (An) and propidium iodide (PI). The analysis was performed with IDEAS software version 6.2 (Amnis/MerckMillipore, Darmstadt, Germany) as described previously [55].

### Western blot analysis

1.25 * 10^6^ HeLa-CD95, HeLa-CD95-C8-KO cells after transfection with procaspase-8a constructs or HT29 cells were seeded into each well of 6-well plates one day prior to treatment. 2.5 * 10^6^ SKW 6.4 cells were prepared on the day of experiment. Cells were harvested, washed with PBS and lysed for 30 min on ice in lysis buffer (20 mM Tris HCl, pH 7.4, 137 mM NaCl, 2 mM EDTA, 10% glycerine, 1% Triton X-100, Protease Inhibitor mix (Roche, Mannheim, Germany)) and subjected to Western Blot analysis. SDS-PAGE was performed with 10% SDS gels. The TransBlot Turbo system (Biorad, Hercules, USA) was used to blot the gels onto nitrocellulose membranes. Blots were blocked with 5% non-fat dried milk in PBS with 0.05% Tween20 for one hour. Washing steps were performed with PBS-Tween three times for 5 min. Incubation with primary antibodies was performed over night at 4 °C in PBS-T. HRP-coupled isotype specific secondary antibodies were incubated for 1 hour at room temperature in 5% non-fat dried milk. This was followed by detection with LuminataForte (Merck Millipore, Darmstadt, Germany) and ChemiDoc imaging system (Biorad, Hercules, USA).

### Silver staining

Silver staining was performed in accordance with the protocols published before [56, 57].

### Immunoprecipitation (IP)

Anti-Caspase-8-, anti-SYM-10-, anti-MMA- and anti-WD45-IPs were performed from 2 * 10^6^ / 10 mL HeLa-CD95-C8-KO cells after transfection with caspase-8a constructs or from 2 * 10^7^ / 10 mL SKW 6.4 cells after 1 000 ng/mL (HeLa-CD95-KO cells) or 250 ng/mL (SKW 6.4) CD95L, CD95L/EPZ or EPZ stimulation. Stimulation was stopped by adding 10 mL cold PBS. Cells were centrifuged for 5 min at 500 x g and washed once with cold PBS. Cells were lysed in 500 µL lysis buffer for 30 min on ice and subsequently centrifuged for 15 min at 14 600xg. 50 µL supernatant was used as input control. The remaining supernatant of all samples was adjusted to the same protein concentration and used for IPs. 2 µg of anti-Caspase-8 antibody or 2 µg of anti-SYM-10 antibody or anti-MMA antibody were added to the lysates and incubated for 12 hours at 4 °C. Subsequently, 10 µL Sepharose-A beads (Abcam, UK) were added, which was followed by the incubation for two hours at 4 °C. For beads-only pulldown, which was used to control unspecific binding, 10 µL Sepharose-A beads (Abcam, UK) were added to the samples without antibodies, which was followed by the incubation for two hours at 4 °C. After incubation, IPs were washed six times with PBS. Samples were subjected to Western Blot analysis.

### Denaturing immunoprecipitation (Den-IP)

Denaturating IPs (Den-IP) were performed by adding 1% SDS after lysing the cells and subsequently heating the samples for 5 min at 95 °C. This was followed by IPs as described above.

### Co-immunoprecipitation (co-IP)

Anti-Caspase-8-co-immunoprecipitation (co-IP), anti-PRMT5-co-IP and anti-CD95-co-IP (CD95-co-IP) were done according to the manufacturer’s instructions via immobilization of the antibodies at the Protein G sepharose beads (Pierce™ Co-Immunoprecipitation Kit; Thermo Fisher Scientific Inc, Waltham, MA, USA).

### Immunoprecipitation and mass spectrometry analysis

Analysis of Caspase-8 and DISC-binding partners were performed by co-IPs. Anti-caspase-8- and anti-APO-1 antibodies were covalently coupled to sepharose beads using Pierce™ Co-Immunoprecipitation according to manufacturer’s instructions (Thermo Fisher Scientific Inc, Waltham, MA, USA). Beads were incubated with total cell lysates of SKW 6.4 cells for 15 min upon 166 ng/mL CD95L treatment or without. The IPs were analysed by nanoLC-tandem mass spectrometry as previously described before [58].

The statistical significance of obtained data was calculated with a one-sample bootstrap method [59], where the 95% confidence intervals were calculated to determine if the scores of methylome proteins were significantly higher from the mean value of other hits. The scipy.stats.bootstrap Python module was used for this purpose with 10 000 resamples.

### Cellular fractionation

2.5 * 10^6^ HeLa-CD95-C8-KO cells after transfection with procaspase-8a constructs were seeded one day before treatment. 5 * 10^6^ of SKW 6.4 cells were taken on the day of treatment. Cells were stimulated with CD95L or left untreated. All centrifugation steps in the following fractionation were performed at 17 000 x g. A swelling step was performed in swelling buffer (10 mM HEPES pH 7.6, 10 mM KCl, 2 mM MgCl_2_, 0.1 mM EDTA, Protease Inhibitor cocktail) for 5 min followed by addition NP-40 (Thermo Fisher Scientific Inc., USA) (0.4% NP-40 for SKW 6.4; 0.6% NP-40 for HeLa-CD95-C8-KO cells after transfection with caspase-8a constructs) for 1 min. Centrifugation for 1 min was used for a separation of the cytoplasmic fraction. Remaining pellet was resuspended in a 500 µl swelling buffer and centrifuged for 15 seconds. The pellet was incubated for 30 min with 40 µL ‘nucleus buffer’ (50 mM HEPES pH 7.8, 50 mM KCl, 300 mM NaCl, 0.1 mM EDTA, 10% Glycerol, Protease Inhibitor cocktail), resuspended every 10 min and centrifuged for 5 min. The nuclei-containing supernatant and the rest-pellet were separated. The samples were subjected to Western Blot analysis.

### Statistics

GraphPad Prism (Version 8.3.0) software was used to calculate and perform statistic tests. The whole data-set of experiments was analysed by ordinary ONE-WAY-ANOVA test. Afterwards comparing specific data sets with each other were done by Tukey-Test. Experiments with two data-sets were analysed by unpaired student t-test. *p*-values are based on the following pattern: (ns / not significant; *p* > 0.05; * significant; *p* < 0.05; ** significant; *p* < 0.01; *** significant; *p* < 0.001; **** significant; *p* < 0.0001).

### Supplementary information

Legends to supplementary Figures

Supplementary Figures

## Data Availability

All data generated or analysed during this study are included in this published article and its supplementary information files.
