# Supplementary material for: Arginine methylation of caspase-8 controls life/death decisions in extrinsic apoptotic networks
Source: Oncogene. 2024 May 10;43(25):1955–71. doi: 10.1038/s41388-024-03049-6 (PMC11178496; doi:10.1038/s41388-024-03049-6)
Supplement: Supplementary file 1 — Legends to supplementary Figures [file 41388_2024_3049_MOESM1_ESM.docx]

**Legends to supplementary Figures**

**Supplementary Figure 1. Mass spectrometry analysis of co-immunoprecipitations**

**(A)** Mass spectrometry identification of PRMT5 unique peptides in caspase-8 co-Immunoprecipitations (caspase-8-co-IP) and CD95-co-IPs. Shown are PRMT5 peptides identified in the different co-IPs. Colours: grey= untreated Casp.8-IP; light yellow = 166 ng/ml CD95L (1 h) treated Caspase-8-IP; blue = untreated CD95-IP; yellow = 166 ng/ml CD95L (1 h) treated CD95-IP. Abbreviations: Casp.8-IP = Caspase-8-IP; IP = Immunoprecipitation. **(B, C)** Silver-stained gels used for mass spectrometry analysis. The co-IPs were loaded together with total cellular lysates on the gels, which were silver stained and cut into individual slices according to molecular weight, followed by proteomic analysis. The co-IPs were loaded together with total cellular lysates on the gels, which was followed by their silver staining and cutting the gel into individual slices according to molecular weight, followed by proteomic analysis. **(D)** SKW 6.4 cells were treated with 200 ng/ml CD95L for indicated time intervals. Cytosolic and nuclear fractions were prepared after treatment. The analysis of EndoG, PARP1 and actin was used as a fractionation control. The fractionation was analysed by Western Blot. One representative fractionation out of two independent experiments is shown.

**Supplementary Figure 2. Caspase-8 is co-immunoprecipitated with PRMT5 and WD45**

**(A-D)** SKW 6.4 cells were treated with indicated concentrations of CD95L for the indicated time intervals.Caspase-8-co-IP **(A)**, Caspase-8-IP / Parkin-IP **(B)**, PRMT5-co-IP **(C)** and WD45-IP **(D)** were performed. IPs and Lysate control (Input) were analysed by Western Blot. **(B)** Parkin-IP was performed as an isotype control IP. One representative experiment out of three independent ones is shown. Abbreviations: IP, immunoprecipitation; B, beads-only pulldown; BC, beads-only pulldown after CD95L treatment; s.e., short exposure; l.e., long exposure.

**Supplementary Figure 3. Selection of the concentration of procaspase-8a-WT for transfection**

HeLa-CD95-C8-KO cells transfected with pcDNA3 (vector), procaspase-8a-WT-pcDNA3 (procaspase-8a-WT) as well as non-transfected (KO) were analysed by Western Blot with the indicated antibodies. Different concentrations of procaspase-8a-WT plasmids were used for transfections. The parental HeLa-CD95 cells are loaded on the same gel as a control for the endogenous expression. One representative experiment out of three independent experiments is shown.

**Supplementary Figure 4. Caspase-8 mutations reduce CD95L-induced caspase cascade**

**(A-E)** HeLa-CD95-C8-KO cells transfected with pcDNA3 (vector), proaspase-8a-R233H-pcDNA3 (R233H), procaspase-8a-R435Q-pcDNA3 (R435Q) and procaspase-8a-WT-pcDNA3 (WT) as well as non-transfected (KO) were treated or not with indicated concentrations of CD95L for the indicated time. **(A,B)** Cells were treated with 500 ng/mL of CD95L for two hours (h) **(A)** and for three hours **(B)** Caspase-Glo® 8 Assay **(A)** and Caspase-Glo® 3/7 Assay **(B)** respectively. **(C)** Cells were treated with indicated concentration of CD95L for 3 h, lysed and subsequently analysed by Western Blot. Protein amount was normalized against loading control actin. One representative experiment out of three is shown. **(D)** Cell viability was captured by measuring ATP levels using CellTiter-Glo® Luminescent Cell Viability Assay. Representative Western Blot controls of transfection efficiency are shown in the lower part of the panels **(A,B,D)** determined Caspase-8 activities. **(E)** Cells were stained by Annexin-V-FITC (An) and Propidium Iodide (PI) and then analysed by Imaging Flow Cytometry. The amount of An and An/PI positive cells after CD95L treatment is shown**.** Mean and standard deviation are shown for three independent experiments. Statistical analysis was carried out by ordinary ONE-WAY ANOVA and followed Tukey-test (ns; not significant; ** significant; p<0.01; *** significant; p<0.001); Abbreviations: s.e., short exposure; l.e., long exposure; An, Annexin V-FITC; PI, Propidium Iodide.

**Supplementary Figure 5. Caspase-8 shows no evidence of mono-methylation (MMA)**

**(A, B)** SKW 6.4 cells were treated with CD95L for the indicated concentration and time. **(A)** caspase-8-co-IP or denaturating Caspase-8-IP (Den-C8-IP) were performed and analysed with anti-MMA and anti-caspase-8 Western Blot. Heavy chain of antibody is marked with IgG_H_. The position of expected signal is indicated with the dashed line. **(B)** MMA-IP was performed and analysed by Western Blot with the indicated antibodies. One representative experiment out of three is shown. Input, IPs and bead-only control are shown. Heavy chain of antibody is marked with IgG_H_. Abbreviations: M, marker.

**Supplementary Figure 6. Pharmacological inhibition of methylation blocks CD95L-induced caspase activity**

**(A)** SKW 6.4 cells were treated with indicated amount of CD95L and/or AMI-5 for indicated time points and concentrations. Caspase-3/7 activity was determined by Caspase-Glo^®^ 3/7 Assay. **(B)** HeLa–CD95 cells were transfected with PRMT5 siRNA or control siRNA for 24 hours. Cells were treated with indicated concentrations of CD95L for six hours and cell viability was captured by measuring ATP levels using CellTiter-Glo® Luminescent Cell Viability Assay. **(C)** Difference between the values of treated and untreated reduction of cell viability from (B) is shown for the indicated transfection. Mean and standard deviation are shown for three independent experiments. Statistical analysis was carried out by ordinary ONE-WAY ANOVA with Tukey-test (** significant; p<0.01; **** significant; p<0.0001).
