# Supplementary material for: Arginine methylation of caspase-8 controls life/death decisions in extrinsic apoptotic networks
Source: Oncogene. 2024 May 10;43(25):1955–71. doi: 10.1038/s41388-024-03049-6 (PMC11178496; doi:10.1038/s41388-024-03049-6)
Supplement: Supplementary file 2 — Supplementary Figures [file 41388_2024_3049_MOESM2_ESM.pptx]

## Slide 1
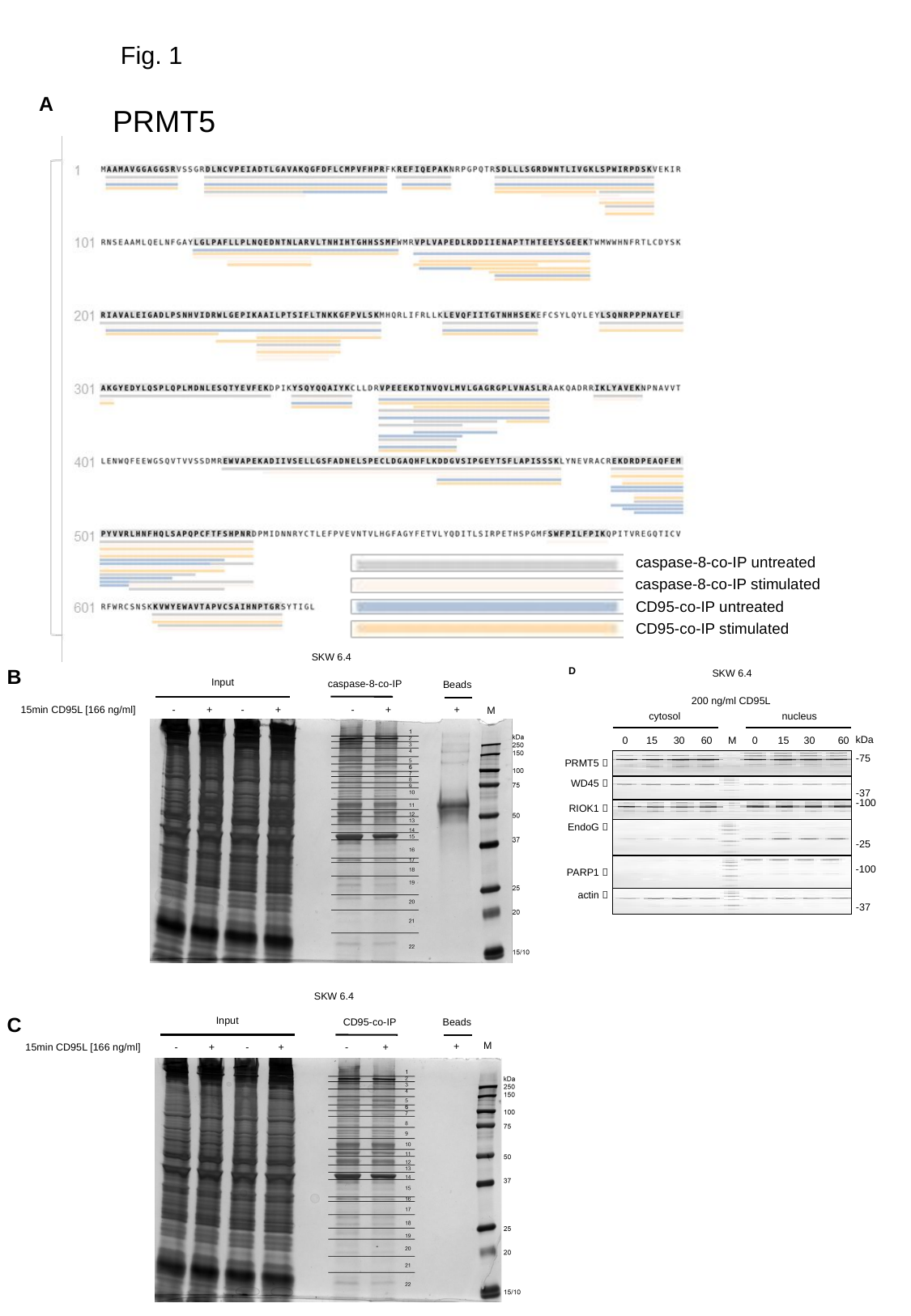

Fig. 1
A
PRMT5
caspase-8-co-IP untreated
caspase-8-co-IP stimulated
CD95-co-IP untreated
CD95-co-IP stimulated
SKW 6.4
B
D
SKW 6.4
Input
caspase-8-co-IP
Beads
200 ng/ml CD95L
 15min CD95L [166 ng/ml]
 -
+
 -
+
 -
+
+
M
cytosol
nucleus
kDa
0
15
30
60
M
0
15
30
60
-75
PRMT5 
WD45 
-37
-100
RIOK1 
EndoG 
-25
-100
PARP1 
actin 
-37
SKW 6.4
C
Input
Beads
CD95-co-IP
M
+
 15min CD95L [166 ng/ml]
 -
+
 -
+
 -
+

## Slide 2
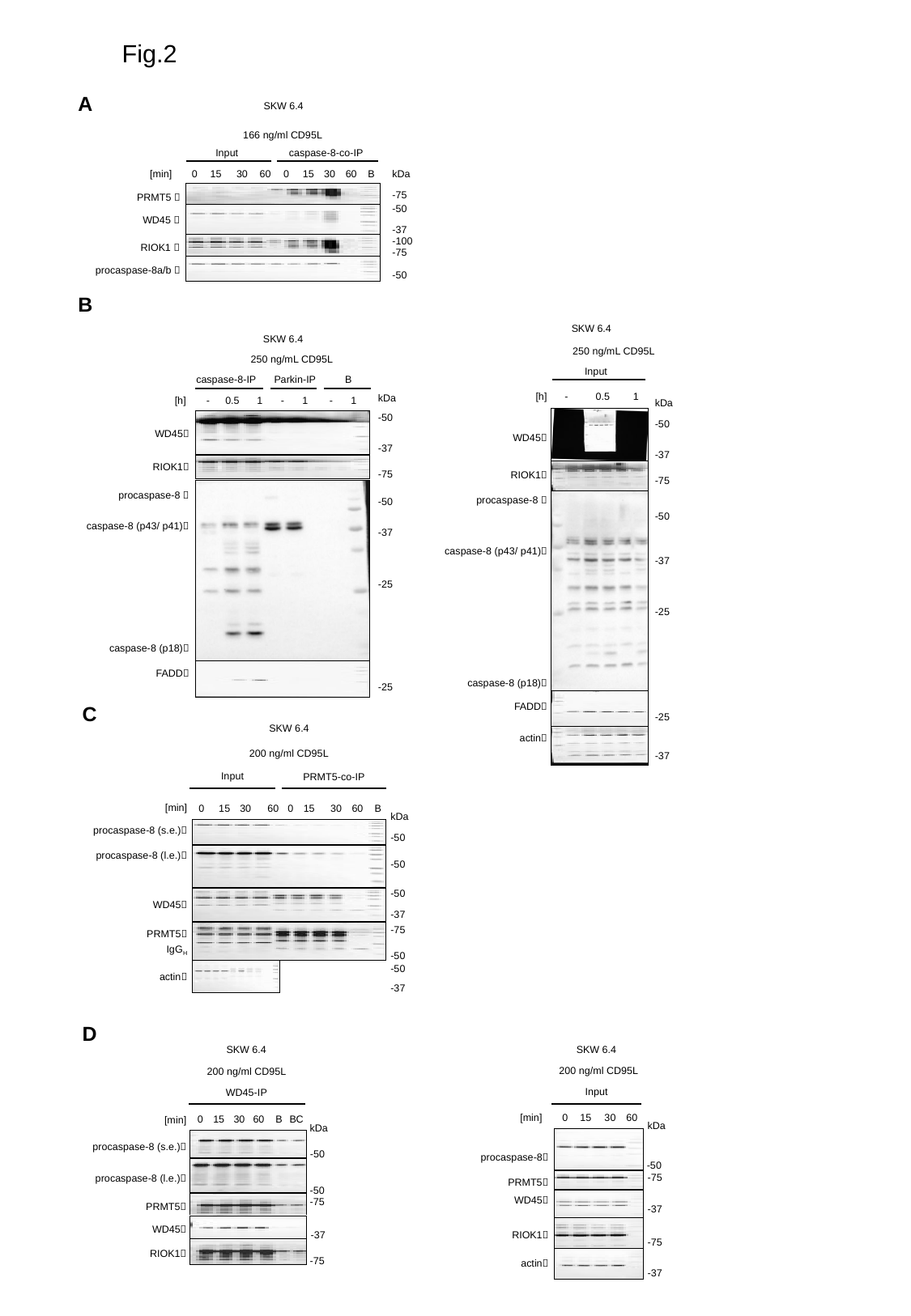

Fig.2
A
SKW 6.4
166 ng/ml CD95L
Input
caspase-8-co-IP
[min]
0
15
30
60
0
15
30
60
B
kDa
-75
PRMT5 
-50
WD45 
-37
-100
RIOK1 
-75
procaspase-8a/b 
-50
B
SKW 6.4
SKW 6.4
250 ng/mL CD95L
250 ng/mL CD95L
caspase-8-IP
Parkin-IP
B
kDa
[h]
-
0.5
1
-
1
-
1
-50
WD45
-37
RIOK1
-75
procaspase-8 
-50
caspase-8 (p43/ p41)
-37
-25
caspase-8 (p18)
FADD
-25
Input
[h]
-
0.5
1
kDa
-50
WD45
-37
RIOK1
-75
procaspase-8 
-50
caspase-8 (p43/ p41)
-37
-25
caspase-8 (p18)
FADD
C
-25
SKW 6.4
actin
200 ng/ml CD95L
-37
Input
PRMT5-co-IP
[min]
0
15
30
60
0
15
30
60
B
kDa
procaspase-8 (s.e.)
-50
procaspase-8 (l.e.)
-50
-50
WD45
-37
-75
PRMT5
IgGH
-50
-50
actin
-37
D
SKW 6.4
SKW 6.4
200 ng/ml CD95L
Input
[min]
0
15
30
60
kDa
-50
-75
-37
-75
-37
procaspase-8
PRMT5
WD45
RIOK1
actin
200 ng/ml CD95L
WD45-IP
0
15
30
60
B
BC
[min]
kDa
procaspase-8 (s.e.)
-50
procaspase-8 (l.e.)
-50
-75
PRMT5
WD45
-37
RIOK1
-75

## Slide 3
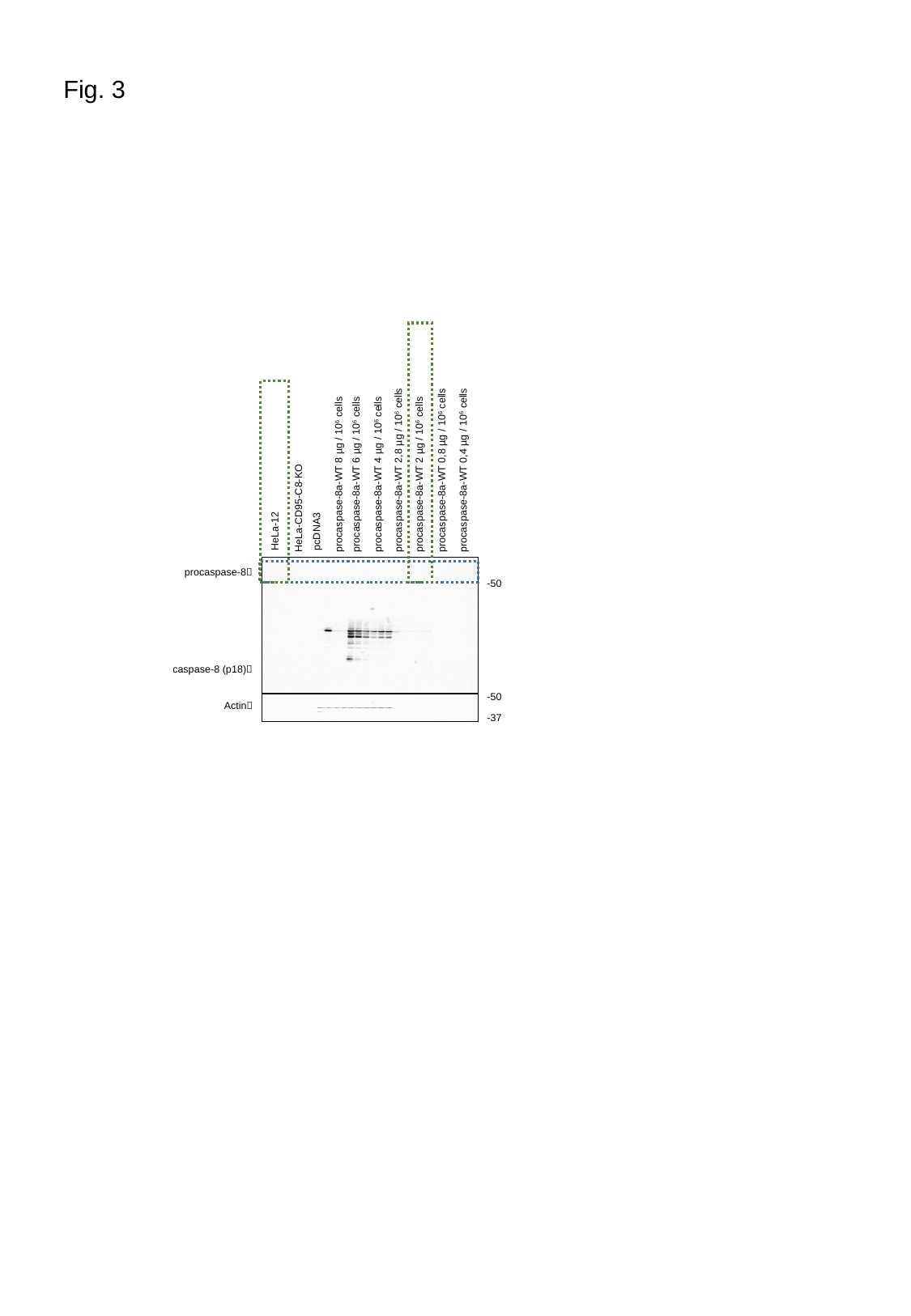

Fig. 3
procaspase-8a-WT 2 µg / 106 cells
procaspase-8a-WT 4 µg / 106 cells
procaspase-8a-WT 2,8 µg / 106 cells
procaspase-8a-WT 0,8 µg / 106 cells
procaspase-8a-WT 0,4 µg / 106 cells
procaspase-8a-WT 8 µg / 106 cells
procaspase-8a-WT 6 µg / 106 cells
HeLa-CD95-C8-KO
HeLa-12
pcDNA3
procaspase-8
-50
caspase-8 (p18)
-50
Actin
-37

## Slide 4
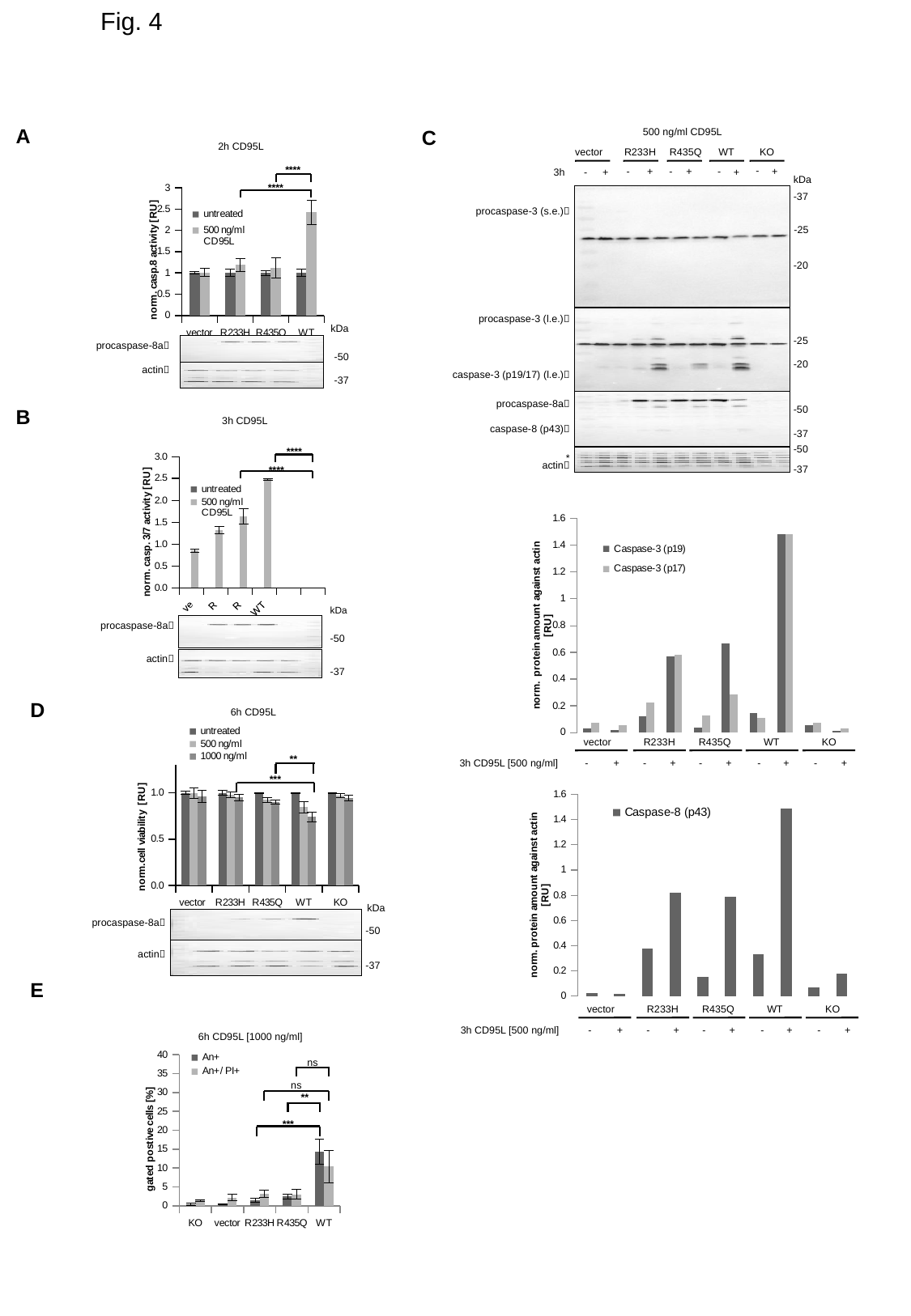

Fig. 4
A
C
500 ng/ml CD95L
2h CD95L
vector
R233H
R435Q
WT
KO
### Chart
| Category | untreated | 500 ng/ml CD95L |
|---|---|---|
| vector | 1.0 | 1.0100795269183185 |
| R233H | 1.0 | 1.1891020885917394 |
| R435Q | 1.0 | 1.122465925311922 |
| WT | 1.0 | 2.424425271520428 |****
-
+
+
+
-
-
-
+
3h
-
+
kDa
****
-37
procaspase-3 (s.e.)
-25
-20
procaspase-3 (l.e.)
kDa
-25
procaspase-8a
-50
-20
actin
caspase-3 (p19/17) (l.e.)
-37
procaspase-8a
-50
B
3h CD95L
caspase-8 (p43)
-37
### Chart
| Category | untreated | 500 ng/ml CD95L |
|---|---|---|
| vector | 1.0 | 0.8532644478655773 |
| R233H | 1.0 | 1.3189494589023665 |
| R435Q | 1.0 | 1.6350649363973526 |
| WT | 1.0 | 2.478011588646088 |-50
****
*
actin
-37
****
### Chart
| Category | Caspase-3 (p19) | Caspase-3 (p17) |
|---|---|---|
| Vektor control | 0.03285697571791572 | 0.07120828841804669 |
| Vektor 500 ng/ml | 0.01651015969016924 | 0.052380194477918494 |
| R233H control | 0.12062801635363192 | 0.22182840982447513 |
| R233H 500 ng/ml | 0.568737 | 0.5813 |
| R435Q control | 0.03889434806699177 | 0.12567691257457902 |
| R435Q 500 ng/ml | 0.6681103397052689 | 0.28185867276932014 |
| WT control | 0.14441268650470118 | 0.10679554465727775 |
| WT 500 ng/ml | 1.484787608556534 | 1.484787608556534 |
| KO control | 0.05687314047646489 | 0.07118586911515493 |
| KO 500 ng/ml | 0.012242234642322459 | 0.03327509505645454 |vector
R233H
R435Q
WT
KO
 3h CD95L [500 ng/ml]
-
+
-
+
-
+
-
+
-
+
kDa
procaspase-8a
-50
actin
-37
D
6h CD95L
### Chart
| Category | untreated | 500 ng/ml | 1000 ng/ml |
|---|---|---|---|
| vector | 1.0 | 0.9964335745718832 | 0.9641603166186262 |
| R233H | 1.0 | 0.9792235799075913 | 0.9498235761626592 |
| R435Q | 1.0 | 0.9230149939031826 | 0.9035334221539394 |
| WT | 1.0 | 0.8445436424159164 | 0.7416691467824496 |
| KO | 1.0 | 0.9706277813414458 | 0.9455449885233188 |**
***
### Chart
| Category | Caspase-8 (p43) |
|---|---|vector
R233H
R435Q
WT
KO
3h CD95L [500 ng/ml]
-
+
-
+
-
+
-
+
-
+
kDa
procaspase-8a
-50
actin
-37
E
6h CD95L [1000 ng/ml]
### Chart
| Category | An+ | An+/ PI+ |
|---|---|---|
| KO | 0.5032 | 1.405 |
| vector | 0.46083333333333326 | 2.2236 |
| R233H | 1.5331333333333335 | 3.2602999999999995 |
| R435Q | 2.4648333333333334 | 3.029133333333333 |
| WT | 14.390799999999999 | 10.4647 |ns
ns
**
***

## Slide 5
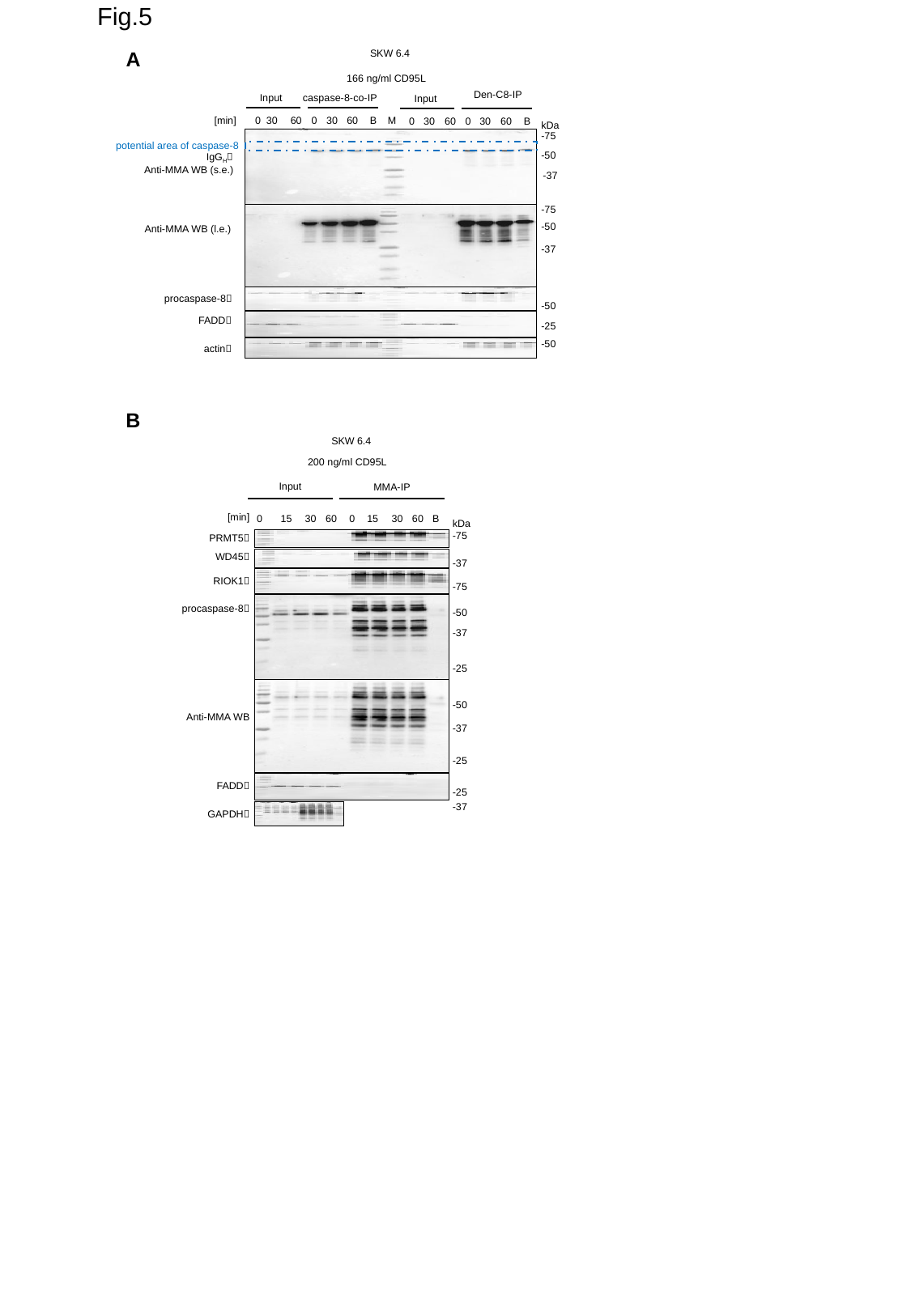

Fig.5
A
SKW 6.4
166 ng/ml CD95L
Den-C8-IP
Input
caspase-8-co-IP
Input
[min]
0
30
60
0
30
60
B
M
0
30
60
0
30
60
B
kDa
-75
-50
Anti-MMA WB (s.e.)
-37
-75
-50
Anti-MMA WB (l.e.)
-37
procaspase-8
-50
FADD
-25
-50
actin
potential area of caspase-8
IgGH
B
SKW 6.4
200 ng/ml CD95L
Input
MMA-IP
[min]
0
15
30
60
0
15
30
60
B
kDa
-75
PRMT5
WD45
-37
RIOK1
-75
-50
-37
-25
-50
 Anti-MMA WB
-37
-25
FADD
-25
-37
GAPDH
procaspase-8

## Slide 6
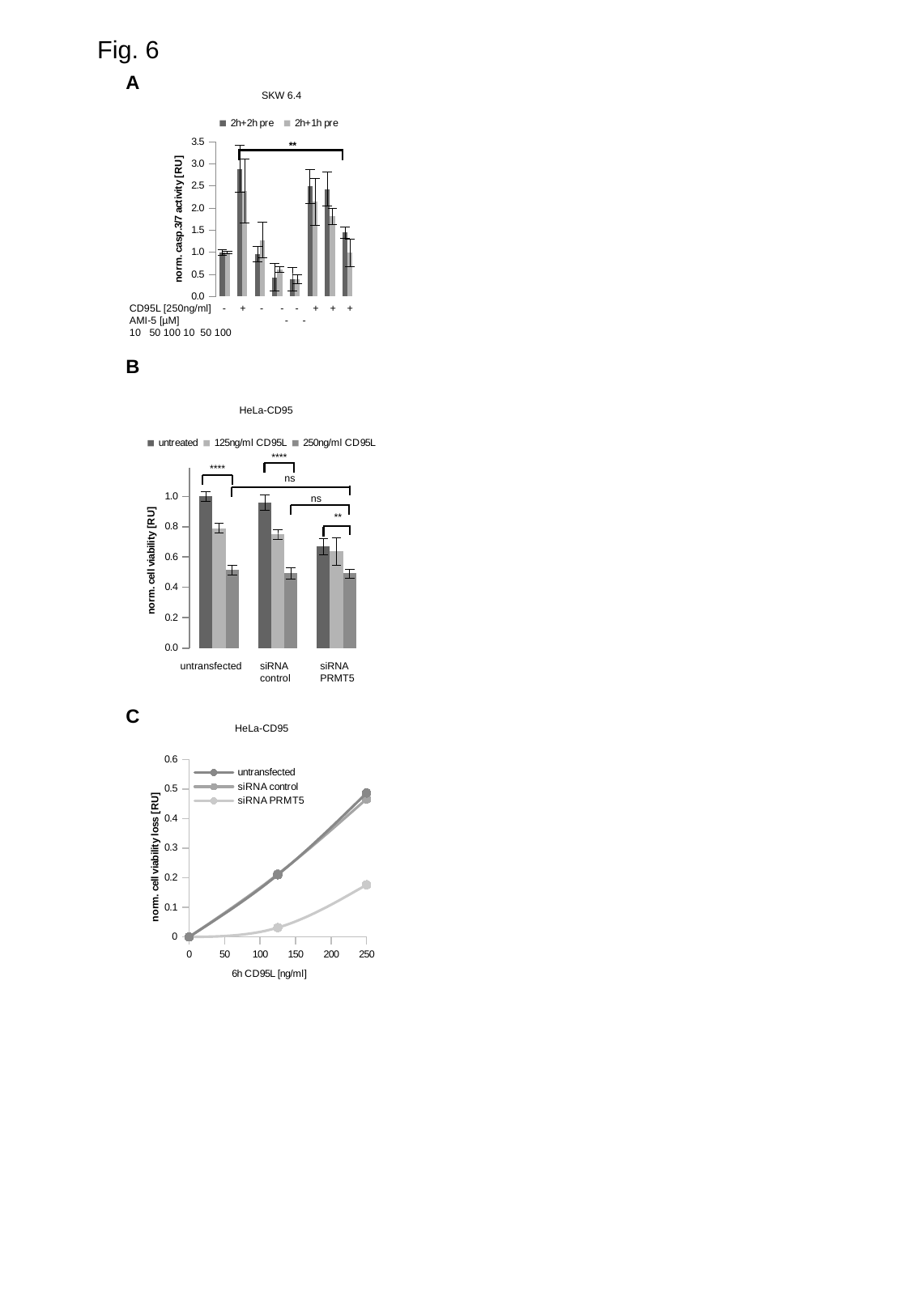

Fig. 6
A
SKW 6.4
### Chart
| Category | 2h+2h pre | 2h+1h pre |
|---|---|---|
| unstimuliert | 1.0 | 1.0 |
| CD95 | 2.8891136911336304 | 2.3915155674124997 |
| 10µM AMI5 | 0.966551672129885 | 1.2779582828166418 |
| 50µM AMI5 | 0.4364714693401513 | 0.6158409099587758 |
| 100µM AMI5 | 0.3957611517169101 | 0.39194564521721104 |
| CD95+10µM AMI5 | 2.4913197957408024 | 2.1478849056572753 |
| CD95+50µM AMI5 | 2.430411035377702 | 1.8227534189995025 |
| CD95+100µM AMI5 | 1.446668597082547 | 0.9968579475777412 |**
CD95L [250ng/ml] - + - - - + + +
AMI-5 [µM] 	 - -	 10 50 100 10 50 100
B
HeLa-CD95
### Chart
| Category | untreated | 125ng/ml CD95L | 250ng/ml CD95L |
|---|---|---|---|
| untransfected | 1.0 | 0.7898670703295751 | 0.5135764849871812 |
| siRNA control | 0.9606320580593287 | 0.7484092454248042 | 0.4942685967859967 |
| siRNA PRMT5 | 0.6686406023356207 | 0.6370573938063343 | 0.49223900035961515 |****
****
ns
ns
**
siRNA
PRMT5
siRNA
control
untransfected
C
HeLa-CD95
### Chart
| Category | untransfected | siRNA control | siRNA PRMT5 |
|---|---|---|---|
